# Modulation of NF-κB and cytokine signaling by *Cordia dichotoma* and *Cordia myxa* attenuates methotrexate-induced pulmonary inflammation and oxidative injury in rats

**DOI:** 10.3389/fphar.2026.1836019

**Published:** 2026-06-26

**Authors:** Nermine M. Mohammed, Hoda M. Hassan, Fatma A. Moharram, Elsayed K. El-Sayed, Asmaa A. Ahmed, Mohamad Elbaz, Merhan E. Ali, Chia-Ying Lin, Kuei-Hung Lai, Shahenda Mahgoub, Hanan G. Sary, Heba T. Khazaal

**Affiliations:** 1 Department of Pharmacognosy, Faculty of Pharmacy, Capital University (Formerly Helwan University), Cairo, Egypt; 2 Department of Pharmacology and Toxicology, Faculty of Pharmacy, Capital University (Formerly Helwan University), Cairo, Egypt; 3 Department of Pathology, Faculty of Veterinary Medicine, Cairo University, Giza, Egypt; 4 School of Food Safety, Taipei Medical University, Taipei, Taiwan; 5 School of Nutrition and Health Sciences, Taipei Medical University, Taipei, Taiwan; 6 Graduate Institute of Pharmacognosy, College of Pharmacy, Taipei Medical University, Taipei, Taiwan; 7 Program in Clinical Drug Development of Herbal Medicine, College of Pharmacy, Taipei Medical University, Taipei, Taiwan; 8 Traditional Herbal Medicine Research Center, Taipei Medical University Hospital, Taipei, Taiwan; 9 Department of Biochemistry and Molecular Biology, Faculty of Pharmacy, Helwan University, Cairo, Egypt; 10 Biochemistry Department, Faculty of Pharmacy, King Salman International University, Ras Sudr, Egypt; 11 Department of Pharmacognosy, Faculty of Pharmacy, Ain-Shams University, Cairo, Egypt; 12 Department of Pharmaceutical Chemistry, College of Pharmacy, Kuwait University, Kuwait

**Keywords:** *Cordia dichotoma*, *Cordia myxa*, lung inflammation, methotrexate, oxidative stress, TGF-β1/α-SMA signaling

## Abstract

**Background:**

Methotrexate (MTX) is a widely used anticancer and immunosuppressive agent, but its clinical use is limited by lung injury associated with oxidative stress, inflammation, and fibrosis. Leaves of *Cordia dichotoma* and *Cordia myxa* traditionally have been used for respiratory disorders, suggesting potential anti-inflammatory benefits.

**Objective:**

To characterize the chemical profile of defatted aqueous methanol extracts (DAME) of *C. dichotoma* and *C. myxa* leaves and evaluate their protective effects against MTX-induced pulmonary inflammation and injury.

**Methods:**

Extracts were analyzed by HPLC/MS. Rats were divided into control, MTX, *Cordia*-pretreated (250 or 500 mg/kg), and dexamethasone groups. Lung tissues were assessed for oxidative stress (MDA, GSH, SOD), inflammatory mediators (TNF-α, IL-1β, IL-6), NF-κB activation, fibrotic markers (TGF-β1, α-SMA, hydroxyproline), and histopathology. *In vitro*, RAW 264.7 macrophages were used to assess cytotoxicity, nitric oxide production, and NF-κB modulation.

**Results:**

HPLC/MS identified 81 and 29 metabolites in *C. dichotoma* and *C. myxa*, respectively, mainly phenolic acids and flavonoids. MTX induced oxidative and inflammatory damage, with elevated cytokine levels and NF-κB activation. Pretreatment with both extracts, particularly *C. dichotoma* (500 mg/kg), significantly restored antioxidant balance, suppressed TGF-β1/α-SMA signaling, and preserved lung architecture. *In vitro*, the extracts inhibited LPS-induced NO release and NF-κB activation, indicating direct macrophage-modulating activity.

**Conclusion:**

Phenolic-rich extracts of *C. dichotoma* and *C. myxa* exert anti-inflammatory, antioxidant, and anti-fibrotic effects by modulating macrophage-driven inflammation and restoring the pulmonary microenvironment, supporting their potential as a natural remedy for inflammation-associated lung injury.

## 1 Introduction

Methotrexate (MTX) is classified as a folic acid antagonist. It is widely used to treat various cancers, including leukemia, lung cancer, and breast cancer, as well as solid tumors. It is also used to manage inflammatory diseases such as rheumatoid arthritis and psoriasis (Ali et al., 2022). Chemotherapeutic drugs, including MTX, have significant side effects that may lead to poor patient adherence. These side effects affect many organs, including the kidneys, liver, brain, and lungs (Jakubovic et al., 2013; Liu et al., 2025). Among these toxicities, pulmonary injury is a major concern, with MTX use linked to lung fibrosis (Fragoulis et al., 2019). Lung fibrosis is a pathological condition in which healthy lung tissue is replaced by mesenchymal cells and extracellular matrix (Zaki et al., 2021). The development of MTX-induced lung fibrosis involves interconnected mechanisms of oxidative stress, inflammation, and fibroblast activation. Excessive generation of reactive oxygen species (ROS) represents an early event that activates redox-sensitive transcription factors, particularly nuclear factor-kappa B (NF-κB) (Zaki et al., 2021; Demir et al., 2024). Activation of NF-κB promotes the transcription of pro-inflammatory cytokines such as tumor necrosis factor-α (TNF-α), interleukin (IL)-1β, and IL-6, thereby amplifying the inflammatory response and tissue injury. Persistent inflammation subsequently stimulates profibrotic pathways, particularly TGF-β1 signaling, leading to myofibroblast activation, α-smooth muscle actin (α-SMA) expression, and extracellular matrix deposition, thereby increasing tissue stiffness. Endothelin-1 (ET-1), a potent vasoconstrictor and bronchoconstrictor, is released from damaged epithelial tissues and contributes to fibrosis (Pulito-Cueto et al., 2023). ET-1 promotes the release of proinflammatory cytokines (Kurt et al., 2015). Furthermore, ET-1, along with profibrotic mediators such as TGF-β1 and tumor necrosis factor-α (TNF-α), sustains myofibroblast activity, leading to increased collagen deposition and α-SMA expression (Duangrat et al., 2023; Holm Nielsen et al., 2019). Accordingly, targeting the NF-κB-dependent inflammatory and fibrotic pathways may represent a significant target to ameliorate inflammation-driven pulmonary fibrosis. Since treatment of lung fibrosis with synthetic drugs can be costly and unsafe due to side effects, researchers are investigating natural, low-cost alternatives with fewer adverse effects. Polyphenols are abundant metabolites found in many plants and are known for their therapeutic activity in human health (Sun et al., 2023). They possess potent antioxidant properties (Cvetković et al., 2024). Research has explored polyphenols as potential anti-inflammatory agents across various inflammatory conditions (Magrone et al., 2019; Liu et al., 2023), since these metabolites can modulate inflammatory signaling pathways through their antioxidant effects. As a result, they are believed to reduce oxidative stress and inhibit the production of proinflammatory mediators by interfering with signal transduction pathways (Singh et al., 2020; Lopez-Corona et al., 2022). The genus Cordia L. (Boraginaceae) comprises approximately 300 species, primarily found in tropical regions worldwide (Oza and Kulkarni, 2017; Raghuvanshi et al., 2022). Several Cordia species, such as *C. dichotoma* and *C. myxa*, are widely used in traditional medicine across South Asia and the Middle East. Decoctions of leaves and/or fruits are used to treat cough, asthma, chest congestion, and other respiratory conditions, as well as fever, inflammation, and wounds (Oza and Kulkarni, 2017). In many traditional medicine systems, the mucilaginous fruits of *C*. *myxa* are taken orally as demulcents and expectorants. At the same time, leaf preparations of *C. dichotoma* are used for inflammatory and gastrointestinal ailments (Oza and Kulkarni, 2017). Notable species include *Cordia dichotoma,* G. Forster, and *C. myxa.* L. *C. dichotoma*, often called Sleshmataka, Lasaura/Lasura, or Mokhate, is a deciduous tree widespread in tropical and subtropical regions (Ibrahim et al., 2019; Oza and Kulkarni, 2017; Singh et al., 2010; Sulieman and El-Newary, 2014). Different parts of the plant have traditionally been used to treat conditions like fever, diarrhea, burning sensations, leprosy, ulcers, tinea, arthralgia, and as a contraceptive (Hussain et al., 2020; Jamkhande et al., 2013; Oza and Kulkarni, 2017; Raghuvanshi et al., 2022; Ahuja et al., 2020). Previous studies have reported that phenolic metabolites were identified from *C. dichotoma* leaves, fruits, seeds, and barks (Bhattacharya and Saha, 2013; Ragasa et al., 2015; El-Newary et al., 2016; Rahman and Akhtar, 2016; Hatware et al., 2018; Mudhubala and Santhi, 2019; Yaermaimaiti, S. et al., 2019; Yaermaimaiti et al., 2021; El-Newary et al., 2022; Raghuvanshi et al., 2022; Hussein et al., 2023; Hussein H. M. et al., 2024; Ibrahim et al., 2024; Joshi et al., 2024). Additionally, from a biological perspective, *C. dichotoma* leaves exhibit antioxidant (Rahman and Akhtar, 2017; Hatware et al., 2018; Joshi et al., 2024); anti-Alzheimer (Hussein H. et al., 2024; Oza and Kulkarni, 2017; Raghuvanshi et al., 2022); neuroprotective (Bhavani et al., 2023); antidiabetic (Madhubala and Santhi, 2021); anthelmintic (Bhaldar et al., 2021); anti-arthritic and thrombolytic (Shembade et al., 2021); gastroprotective and anti-inflammatory (Hatware et al., 2018); anticancer (Rahman and Akhtar, 2017; Rahman and Hussain, 2015); antifertility (Bhattacharya and Saha, 2013; Sharma et al., 2015); and analgesic, antipyretic, and anti-inflammatory activities (Gupta and Kaur, 2015). *C. myxa* L (Syn. *C. obliqua*, *C. crenata*), an edible plant known as the Assyrian plum, Indian cherry, Lasoda, and Lehsua, grows in tropical and subtropical regions of Asia, from the eastern Mediterranean to eastern India, as well as in America and Australia (Oza and Kulkarni, 2017; Tak et al., 2024). Traditionally, it has been used to treat various conditions, including wound healing, demulcent, anthelmintic, diuretic, astringent, emollient, expectorant, hepatoprotective, analgesic, hypoglycemic, anti-inflammatory, laxative, flu, fever, cough, cold, asthma, hypolipidemic, aphrodisiac, and antiulcer (Oza and Kulkarni, 2017; Wani et al., 2023). Chemically, *C. myxa* leaves and fruits have been reported to contain phenolic metabolites (El-Massry et al., 2021; Al-Musawi et al., 2022; Kumar et al., 2022; El-Nashar et al., 2025). From a biological perspective, the leaves exhibit various activities, including hepatoprotective (Afzal et al., 2007), anthelmintic (Rashid et al., 2015; Atia and Al-Bahadyli, 2018), and cognitive dysfunction treatments (Kendir et al., 2022). Furthermore, leaves and fruits show antidiabetic, antioxidant, anti-inflammatory, and analgesic effects (Ranjbar et al., 2013; Abdel-Aleem et al., 2019; Samari et al., 2019; El-Nashar et al., 2025). Although some biological activities have been reported for the leaves of *C. dichotoma* and *C. myxa,* their potential effects against MTX-induced lung toxicity in rats have not been documented. Therefore, in this study, we examine, for the first time, the potential effects of the defatted aqueous methanol extract (DAME) of their leaves on lung toxicity and correlate these effects with their metabolites tentatively identified using HPLC/MS. While previous studies have reported individual antioxidant or anti-inflammatory activities of Cordia species, a comparative and integrated evaluation of *C. dichotoma* and *C. myxa* within the MTX-induced pulmonary injury model remains limited. Considering the traditional use of *C. dichotoma* and *C. myxa* leaf preparations for respiratory and inflammatory issues, we hypothesized that phenolic-rich DAME from these leaves might reduce MTX-induced lung damage. Our study followed the Four Pillars of Best Practice in Ethnopharmacology, emphasizing ethnomedical context, detailed extract analysis, and thorough pharmacological assessment of NF-κB-mediated inflammatory pathways and TGF-β1-driven fibrotic signaling.

## 2 Materials and methods

### 2.1 Plant material

*Cordia dichotoma* G. Forst. (leave, Boraginaceae), Moreover, *Cordia myxa* L.(Boraginaceae) leaves were collected in July 2024 from the Mazhar Botanical Garden in Cairo, Egypt. They were gathered after obtaining permission from the garden authorities, in accordance with the local garden’s guidelines. Collection and identification were performed under the supervision of Dr. Trease Labib, a senior botanist at Mazhar Botanical Garden (Cairo, Egypt), who authenticated the botanical drugs using macroscopic and microscopic morphological criteria and comparison with reference material. Taxonomic identities were confirmed using the International Plant Names Index (IPNI; https://www.ipni.org). The accepted names and their identifiers are *Cordia dichotoma* G.Forst. (IPNI ID 114098-1) and *Cordia myxa* L. (IPNI ID 97109-3). Voucher specimens from each species were deposited in the Pharmacognosy Department, Faculty of Pharmacy, Helwan University (3 Cdi 1/2024; 3 Cmy 3/2024). The plant material used in this research is endotoxin-free.

### 2.2 Chemicals and reagents

Methanol, *n*-hexane, normal saline, buffered formalin, and phosphate buffer were purchased from El Nasr Pharmaceutical Chemicals Co. (Gesr El Suez, Cairo, Egypt). A Unitroxate® vial containing 50 mg/2 mL of Methotrexate (MTX) was obtained from Hikma Pharmaceutical Co., Egypt. Dexamethasone (DEXA) 8 mg/2 mL vials were acquired from Al Amriya Pharmaceutical Industries Co., Egypt.

### 2.3 Preparation of the defatted extract

Leaves of *C. dichotoma* and *C. myxa* (450 g each) were separately extracted with 80% aqueous methanol under reflux (4 × 3 L each), then filtered. The methanol solution evaporated under vacuum at 50 °C. The dry extracts were defatted with *n*-hexane under reflux (3 × 1.5 L each), followed by solvent removal under reduced pressure. Although traditional uses involve preparing aqueous decoctions or infusions from leaves and fruits for demulcent and anti-inflammatory effects (Oza and Kulkarni, 2017), the 80% aqueous methanol DAME used here is specifically optimized to maximize phenolic content. The extract was concentrated and defatted with n-hexane, yielding DAME. While not identical to traditional preparations, it provides a standardized, phenolic-rich fraction for future comparison with traditional aqueous extracts.

### 2.4 Estimation of total phenolic content (TPC)

It was performed using the Folin-Ciocalteu colorimetric method (Najafi et al., 2022; Moharram et al., 2025). Absorbance was measured at 765 nm with a UV-visible spectrophotometer (Shimadzu UV-Vis Spectrophotometer, UV-1900i, Japan). Results were derived from the calibration curve of the standard (gallic acid 50–300 μg/mL) using the following equation: y = 0.0052x + 0.1069 (*R* ^2^ = 0.999).

### 2.5 Estimation of total flavonoid content (TFC)

Colorimetric measurement was performed using AlCl_3_ (El Haggar et al., 2023). Absorbance readings were obtained at 415 nm using a UV-visible spectrophotometer (Shimadzu UV-1900i, Japan). The results were calculated using the calibration curve derived from the standard (quercetin, 50–300 μg/mL), following the equation y = 0.0244x + 0.0642 (R2 = 0.9986).

Results were expressed as milligrams of quercetin equivalent (mg QE/mg of dry weight).

### 2.6 HPLC/MS determination of individual phenolic compounds

The HPLC/MS analysis utilized a Waters Corporation XEVO triple quadrupole (TQD) mass spectrometer (Milford, MA, United States), operating in both negative and positive ion modes. Chromatography was performed on the ACQUITY UPLC BEH C18 column (1.7 µm × 2.1 mm × 50 mm, 28.4 °C) with 0.1% formic acid in water as solvent A and methanol (MeOH) as solvent B. Isocratic elution at a flow rate of 0.2 mL/min run off 32 min, following this program: 10% B for 5 min, 30% B for 10 min, 70% B for 7 min, and 90% B for 10 min. The MS acquisition range covered m/z 50–1000.

### 2.7 Identification of the metabolites

Tentative metabolites identification was based on MS data in both negative and positive ion modes, as well as comparisons with previously identified metabolites from the same plant or family, or with naturally occurring compounds.

### 2.8 Evaluation of the protective effect against lung toxicity induced by MTX

#### 2.8.1 Animals

Female *Sprague-Dawley* rats (170–180 g) were obtained from the breeding facility of the Egyptian Organization for Biological Products and Vaccines in Helwan, Egypt. They were acclimated for 1 week in an animal house maintained at 23 °C ± 2 °C with a 12-h light/dark cycle. During this period, they had free access to water and a standard laboratory pellet diet. Animal care and experiments were approved by the Institutional Animal Care and Use Committee (IACUC) of the Faculty of Pharmacy, Capital University (formerly Helwan University) (Protocol Number: 21 A2024). The sample size was determined based on previous studies using similar experimental models, ensuring sufficient power to detect statistically significant differences in the measured parameters among the tested groups while remaining consistent with ethical considerations for animal use. Humane endpoints were applied throughout the study; animals showing >20% body weight loss, severe lethargy, or respiratory distress were humanely euthanized to minimize suffering.

#### 2.8.2 Study of the acute toxicity

An acute oral toxicity study was conducted to evaluate the safety profile of the DAMEs of *C. dichotoma* and *C. myxa*. Healthy adult rats were administered single oral doses of each extract at increasing concentrations up to 5 g/kg body weight. The animals were observed continuously for the first 24 h for signs of toxicity, behavioral changes, or mortality.

#### 2.8.3 Experimental design

Forty-nine female *Sprague-Dawley* rats were randomly assigned to seven groups (n = 7 each). Group 1 received oral normal saline and served as the control group. Group 2: MTX group: Rats administered MTX orally (14 mg/kg/week, for 2 weeks) (Ali et al., 2022). Groups 3-4: Rats received DAME of *C. dichotoma* 250 and 500 mg/kg, respectively, *p.o*. for 14 days, 1h before MTX. Groups 5-6: Rats received DAME of *C. myxa* 250 and 500 mg/kg, respectively, *p.o*. for 14 days, 1h before MTX. Group 7: Rats received dexamethasone (DEXA) as a standard treatment (0.5 mg/kg/day, i.p.) for 14 days (Fikry et al., 2015), one hour before MTX. The extract doses were determined based on the acute oral toxicity study.

Twenty-four hours following the final dose of DAMEs and DEXA, rats were euthanized *via* cervical dislocation after being anesthetized with ketamine (50 mg/kg) (El-Sayed et al., 2023). Lungs were quickly separated, washed with ice-cold normal saline, and weighed. The lungs were homogenized in phosphate-buffered saline (10% w/v) and then centrifuged at 4,000 rpm for 15 min. The cleared supernatants were stored at −80 °C for biochemical analysis. Additionally, other sections of the lungs were preserved in formalin–saline (10%) for histopathological examinations.

#### 2.8.4 Assessments of oxidative stress markers

MDA, GSH, and SOD levels in lung tissues were measured using commercial kits (Eagle Biosciences, Cat No. LIP39-K01, Amherst, Massachusetts, United States; BioVision, Cat No. K264-100, Milpitas, CA, United States; and Biodiagnostic, Cat No. SD 25 21, Giza, Egypt).

#### 2.8.5 Assessment of inflammatory indicators

A BioLegend ELISA kit was used to determine the TNF-α level in lung tissues (Cat No: 438206, San Diego, CA, United States). ELISA kits from ELK Biotechnology were used to measure IL-1β and IL-6 levels (Cat Nos. ELK1272 and ELK1158, respectively; Hubei, China). An ELISA kit from Fine Test (Cat No: ER1186, Wuhan, Hubei, China) was used to measure NF-κB levels.

#### 2.8.6 Assessment of fibrotic parameters

ELISA kits were utilized to measure the TGF-β1 and hydroxyproline levels in lung tissues. TGF-β1 (FineTest, Cat No: ER1378, Wuhan, Hubei, China) and hydroxyproline (BT LAB, Cat No: EA0040Ra, Jiaxing, Zhejiang, China).

#### 2.8.7 Assessment of ET-1

The level of ET-1 in lung tissues was estimated by the Elabscience ELISA kit (Cat No: E-EL-R1458, Houston, Texas, United States).

#### 2.8.8 Estimation of protein content

The protein content in the tissue was estimated by colorimetric analysis using the Bradford (1976) method.

#### 2.8.9 Histopathological examination and Masson’s trichrome staining of lung tissues

Lung tissue samples were dissected and fixed in 10% neutral buffered formalin (HT501128, Sigma-Aldrich, Louis, MO, United States) for 72 h. They were then subjected to a series of ethanol washes, cleared with xylene, and embedded in Paraplast (39602012, Leica Biosystems, Nussloch, Germany). 4-μm-thick sections were cut with a rotary microtome (RM2255, Leica Biosystems) and mounted on glass slides for analysis of the pulmonary parenchyma. These sections were stained with Hematoxylin and Eosin (Cat No: ab245880, abcam, Cambridge, United Kingdom) for general histology, followed by Masson’s trichrome (Cat No: ab150686, abcam, Cambridge, United Kingdom) to emphasize collagen fibers. An experienced histologist examined all sections in a blinded manner. All fixation and staining procedures adhered to standard protocols outlined by Culling (2013).

#### 2.8.10 Immunohistochemical staining of lung tissues to estimate α-SMA

Following the manufacturer’s protocol, deparaffinized tissue sections (4 μm thick) were treated with 3% H_2_O_2_ for 20 min, washed, and then incubated overnight with anti-α-SMA antibody (Cat No: Ab5694, Abcam Limited, Cambridge, United Kingdom) at a 1:100 dilution. After PBS washing, they were incubated with the HRP-conjugated EnVision secondary antibody (DAKO, Denmark) for 20 min. The sections were subsequently washed with PBS, incubated with diaminobenzidine (DAB) for 10 min, rewashed, counter-stained with hematoxylin, dehydrated, cleared in xylene, and cover slipped for microscopic examination.

#### 2.8.11 Microscopic analysis

Using a Leica Microsystems GmbH Full HD microscopic imaging system controlled by Leica Application software, the examiner histologist randomly selected at least six non-overlapping fields per tissue section of each sample at ×40 magnification. To ensure standardization of tissue evaluation, all fields were selected to include comparable lung regions containing both perivascular and peribronchial structures across all experimental groups. These fields were scanned to measure the relative area percentage of collagen fibers in Masson’s trichrome-stained sections and the area percentage of immunohistochemical expression levels of α-SMA in immunostained sections (Mohamed et al., 2022).

#### 2.9. *In vitro* assay

##### 2.9.1. Cell viability examination

RAW 264.7 cells (5 × 10^4^ cells/mL, 100 µL per well) were cultured in 96-well plates and treated with DC, MC, or celecoxib at concentrations ranging from 4 to 1,000 μg/mL for 24 h. After incubation, 100 µL of MTT solution (0.5 mg/mL) was added, and cells were further incubated for 6 h at 37 °C. The medium was then removed, formazan crystals were dissolved in 100 µL DMSO, and absorbance was measured at 490 nm using a microplate reader.

##### 2.9.2 Inhibition of nitric oxide (NO) and NF-κB production in lipopolysaccharide (LPS)-stimulated RAW 264.7 cells

RAW 264.7 macrophages were seeded at 5 × 10^5^ cells/well in 24-well plates and incubated for 12 h at 37 °C, 5% CO_2_. After removal of the medium, cells were treated with FBS-free DMEM containing CM, CD, celecoxib, or vehicle at a dose equivalent to 25% of the IC_50_ in a final volume of 500 µL. Following 1 h of exposure, cells were stimulated with LPS (1 μg/mL) for 24 h.

Nitrite and NF-κB levels in culture supernatants were quantified using a commercial NO detection kit (ab272517 Nitric Oxide Assay Kit, Japan) and (LSBio Mouse NF-kappaB ELISA Kit, United States). All assays were carried out according to the manufacturers’ protocols, and standard curves with high linearity (*R* ^2^ ≥ 0.99) were generated for each experiment. Absorbance was measured at 540 nm using a bioline elisa reader. Nitrite concentrations were calculated from a sodium nitrite (NaNO_2_) standard curve. The assay performances were monitored by evaluating intra- and inter-assay variability, with coefficients of variation maintained within acceptable ranges (<10% and <15%, respectively).

### 2.10. Statistical analysis

All values are presented as mean ± SE (n = 7). Data were first tested for normality using the Shapiro-Wilk test. Because the data followed a normal distribution, one-way ANOVA with Tukey’s *post hoc* test for multiple comparisons was used. For each comparison, the mean difference, 95% confidence interval (CI), and *p*-value were calculated. Statistical analyses were performed using GraphPad Prism version 8 (GraphPad Software, Inc., La Jolla, CA, United States), and differences were considered statistically significant at *p* < 0.05. All *in vitro* experiments were performed with at least three independent biological replicates.

## 3 Results

### 3.1 Percentage yields of the extracts

Extraction of 450 g of *C. dichotoma and C. myxa* with 80% aqueous methanol yielded 32 g (7.1%) and 41 g (9.1%) from each species, respectively, while the defatted extract (DAME) accounted for 11 g (2.4%) and 14 g (3.1%) for *C. dichotoma* and *C. myxa*, respectively.

### 3.2 Quantification of phenolic and flavonoid contents from two Cordia species

As mentioned earlier, the total phenolic and flavonoid contents in the DAME of the two studied species were measured using colorimetric assays. The results (Table 1) show that *C. dichotoma* (134.42 ± 5.31) has a higher TPC than *C. myxa* (117.80 ± 1.69). Meanwhile, *C. dichotoma* (10.72 ± 0.41) exhibited greater total flavonoid content than *C. myxa* (8.25 ± 0.30). This is the first report on the TFC and TPC of DAME from these two plants, although the TPC and TFC of *C. dichotoma* methanol leaf extract were previously measured (137.9 ± 6.76 mg GAE/g and 22.44 ± 1.21 RE/g, respectively) (Hussein H. et al., 2024). Additionally, the TPC and TFC of *C. myxa* (71.73 ± 2.27 mg GAE/g and 13.85 ± 0.18 RE/g, respectively) were reported by El-Nashar et al. Although the TPC and TFC of the leaves of these two plants have been previously documented, differences between our data and those reports could be due to factors such as collection site and variations in extraction conditions (solvent, time, and temperature) (Ali et al., 2021).

**TABLE 1:** Estimated total phenolic content (TPC) and total flavonoid content (TFC) in the defatted aqueous methanol extract (*DAME*) of *C. dichotoma* and *C. myxa* leaves.

Species	TPC (mg GAE/g)	TFC (mg QE/g)
*C. dichotoma*	134.42 ± 5.31	10.72 ± 0.41
*C. myxa*	117.80 ± 1.69	8.25 ± 0.30

Data represented as mean ± SEM (n = 3).

### 3.3 HPLC/MS analysis for DAME of *C. dichotoma* and *C. myxa* leaves

The HPLC/MS analysis of the DAME from the two Cordia species revealed the presence of 81 secondary metabolites in *C*. *dichotoma* and 29 in *C*. *myxa,* belonging to various classes. These metabolites were tentatively identified in both positive- and negative-ion modes (Table 2; Supplementary Table 1). The chromatograms of the HPLC/MS are shown in Supplementary Figures S1–S4.

**TABLE 2:** HPLC/MS tentative identification of phenolic metabolites from the defatted aqueous methanol extract (DAME) of *Cordia dichotoma* and *Cordia myxa*.

No.	Compounds	MF	RT (min)	Mode of ionization	MW	Observed (*m*/*z*)	CD	CM	Reference
Hydroxybenzoic phenolic acids									
1	Syringic acid	C_9_H_10_O_5_	1.34	[M-H]^−^	198.05	197.1195	+	-	Hussein et al. (2024b)
2	Protocatechuic acid	C_7_H_6_O_4_	1.34	[M-H]^−^	154.12	152.8272	+	-	Al-Musayeib et al. (2011)
3	Vanillic acid	C_8_H_8_O_4_	5.71	[M-H]^−^	168.042	166.9690	+	+	El-Newary et al. (2022)
4	2-Hydroxybenzoic acid	C_7_H_6_O_3_	27.44	[M+H]^+^	138.122	139.0841	+	-	Yaermaimaiti et al. (2021)
Hydroxycinnamic phenolic acid									
5	Latifolicinin C	C_9_H_10_O_4_	0.69	[M-H]^−^	182.175	181.0080	+	+	Yaermaimaiti et al. (2019)
6	*p*-Coumaric acid	C_9_H_8_O_3_	5.71	[M-H]^–^	164.158	163.0250	+	-	Hussein et al. (2024a)
7	Ferulic acid	C_10_H_10_O_4_	7.01	[M-H]^−^	194.18	193.0579	+	-	Hussein et al. (2024a)
8	Rosmarinic acid	C_18_H_16_O_8_	7.19	[M-H]^−^	360.084	359.0526	+	+	El-Nashar et al. (2025)
9	3-*p*-coumaroylquinic acid	C_16_H_18_O_8_	7.67	[M-H]^−^	338.31	337.2144	+	+	Hussein et al. (2024b)
10	Chlorogenic acid	C_16_H_18_O_9_	18.42	[M-H]^−^	354.09	353.2972	+	-	Hussein et al. (2024a)
11	Caffeoylquinic acid	C_16_H_18_O_9_	18.63	[M-H]^−^	354.31	353.3080	+	-	Baran et al. (2024)
12	Caffeyltartaric acid	C_13_H_12_O_9_	19.67	[M-H]^−^	312.23	311.2159	+	-	Zhu et al. (2021)
13	6-*O*-*p*-Coumaroyl-D-glucose	C_15_H_18_O_8_	27.81	[M-H]^−^	326.298	325.2235	+	-	Cao, et al. (2022)
14	*Trans-* cinnamic acid	C_9_H_8_O_2_	30.24	[M-H]^–^ [M+H]^+^	148.158	147.0022 149.9628	+	-	Hussein et al. (2024a)
Phenolic acid derivatives									
15	Syringaldehyde	C_9_H_10_O_4_	1.18	[M-H]^–^	182.173	181.0726	+	-	Hussein et al. (2024b)
16	8*S*-(2)-8-(4-hydroxy-3-metho y benzoyl) - dihydrofuran-8(8′H)-one	C_12_H_12_O_5_	5.71	[M-H]^−^	236.223	235.1958	+	-	Yaermaimaiti et al. (2021)
17	Methyl *p*-hydroxyphenyl lactate	C_10_H_12_O_4_	6.31	[M+H]^+^	196.20	197.1066	+	-	Yaermaimaiti et al. (2021)
18	1-(4-Hydroxyphenyl) propan-1-one	C_9_H_10_O_2_	13.60	[M+H]^+^	150.18	151.6856	+	-	Yaermaimaiti et al. (2021)
19	4-(3-(4-hydroxy-3-methox yphenyl)-3-oxopropoxy)-4-oxobutanoic acid	C_14_H_16_O_7_	14.53	[M-H]^–^	296.272	295.1613	+	-	Yaermaimaiti et al. (2021)
20	3-(4-Hydroxy-3-methoxyphenyl)-3-oxopropyl methyl succinate	C_15_H_18_O_7_	18.30	[M-H]^−^	310.302	309.2224	+	-	Yaermaimaiti et al. (2021)
21	Evofolin B	C_17_H_18_O_6_	22.56	[M+H]^+^	318.32	319.1006	+	-	Yaermaimaiti et al. (2021)
22	Coniferyl aldehyde	C_10_H_10_O_3_	27.91	[M-H]^–^	178.184	177.8468	+	-	Yaermaimaiti et al. (2021)
23	Hydroxybenzaldehyde	C_7_H_6_O_2_	28.44	[M+H]^+^	122.123	123.9877	+	+	Yaermaimaiti et al. (2019)
24	4-Vinylphenol	C_8_H_8_O	28.83	[M+H]^+^	120.148	121.9741	+	-	Bughio, et al. (2024)
25	Protocatechualdehyde	C_7_H_6_O_3_	29.30	[M-H]^–^	138.120	137.0588	+	-	Yaermaimaiti et al. (2021)
26	Catechol	C_6_H_6_O_2_	29.67	[M-H]^−^	110.111	109.7780	+	-	Yaermaimaiti et al. (2021)
Flavonoids									
Flavonol									
27	Isorhamnetin	C_16_H_12_O_7_	1.25	[M-H]^−^	316.058	315.055	+	-	Kuppast et al. (2006)
28	Quercetin-pentoside	C_20_H_18_O_11_	5.03	[M-H]^−^	434.08	433.2385	+	-	Zhang et al. (2018)
29	Rutin	C_27_H_30_O_16_	15.89	[M+H]^+^	610.15	611.4144	+	-	Hussein et al. (2024a)
30	Isorhamnetin-3-*O*-rutinoside	C_28_H_32_O_16_	18.42	[M-H]^−^	624.17	623.3145	+	+	Oza and Kulkarni (2017)
31	Quercetin 4′,7-diglucoside	C_27_H_30_O_17_	18.48	[M+H]^+^	626.51	627.4552	+	-	Kwak et al. (2017)
32	Quercetin 7-glucuronide 3-rhamnoside	C_27_H_28_O_17_	18.63 18.76	[M-H]^–^ [M+H]^+^	624.501	623.3346 625.3190	+	-	Anand et al. (2022)
33	Myricetin rhamnosyl-hexoside	C_27_H_30_O_17_	19.75	[M+H]^+^	626.5169	627.4141	+	-	Sobeh et al. (2018)
34	Quercetin 3-(6″-acetyl-galactoside) 7-rhamnoside	C_29_H_32_O_17_	21.86	[M+H]^+^	652.5542	653.4166	+	+	Soleh Ismail et al. (2021)
35	Quercetin 3-(4″-acetylrhamnoside) 7-rhamnoside	C_29_H_32_O_16_	22.56	[M+H]^+^	636.554	637.3945	+	+	Soleh Ismail et al. (2021)
36	Quercetin 3-(6″-malonyl-glucoside)	C_24_H_22_O_15_	25.16	[M+H]^+^	550.4225	551.5068	+	-	Enkhmaa et al. (2005)
37	Kaempferol-3,7-di-*O*-*α*-*L*-rhamnopyranoside	C_27_H_30_O_14_	26.71	[M+H]^+^	578.518	579.3589	+	-	Nogueira et al. (2013)
38	Aromadendrin	C_15_H_12_O_6_	30.38	[M-H]^−^	288.25	287.0324	+	-	Hussein et al. (2024a)
39	Taxifolin	C_15_H_12_O_7_	31.23	[M-H]^−^	304.254	304.9458	-	+	Roy et al. (2014)
Flavone									
40	Apigenin 7,4′-dimethyl ether	C_17_H_14_O_5_	18.63	[M-H]^−^	298.290	297.2844	+	-	Krishna et al. (2015)
41	Luteolin 7-*O*-diglucuronide	C_27_H_26_O_18_	18.76	[M+H]^+^	638.484	639.3757	+	+	Abu-Lafi et al. (2024)
42	3′,5,6-trihydroxy-3,4′,7,8-tetramethoxyflavone 3-glucoside	C_25_H_28_O_14_	19.46	[M-H]^–^	552.481	551.3143	+	-	Wan et al. (2022)
43	3,7,4` -trimethoxyflavone	C_14_H_16_O_8_	19.67	[M-H]^−^	312.32	311.2159	+	-	Correia Da Silva et al. (2010)
44	Luteolin 7-*O*-(2-apiosyl-6-malonyl)-glucoside	C_29_H_30_O_18_	21.86	[M+H]^+^	666.537	667.3212	+	-	Le et al. (2023)
45	Tricin 7-neohesperidoside	C_29_H_34_O_16_	21.86	[M+H]^+^	638.570	639.3583	+	+	Sirijan et al. (2025)
46	Diosmin	C_28_H_32_O_15_	22.06	[M+H]^+^	608.544	609.5275	+	+	Gerges et al. (2022)
47	5-Hydroxy-3,7,4′-trimethoxyflavone	C_18_H_16_O_6_	22.92	[M-H]^−^	328.316	327.3428	-	+	Oza and Kulkarni (2017)
48	Apigenin-7-*O*-diglucuronide	C_27_H_26_O_17_	23.84	[M+H]^+^	622.485	623.3438	+	+	Inomata et al. (2013)
49	Apigenin 7-*O*-(6″-malonyl-apiosyl-glucoside)	C_29_H_30_O_17_	27.66	[M+H]^+^	650.538	651.5445	+	-	Ali et al. (2021)
50	Luteolin	C_15_H_10_O_6_	29.30	[M-H]^−^	286.24	285.9200	-	+	Raghuvanshi et al. (2022)
Flavanones									
51	Naringenin-7-*O*-*β*-*D*-glucoside	C_21_H_22_O_16_	5.03	[M-H]^−^	434.08	433.2385	+	-	Baran et al. (2024)
52	Hesperidin	C_28_H_34_O_15_	15.89	[M+H]^+^	610.19	611.4144	+	-	Ficarra et al. (1995)
53	Rhusflavanone	C_30_H_22_O_10_	15.99	[M-H]^−^	542.496	541.4384	-	+	Priyadarsani Mandhata et al. (2023)
54	Hesperetin-7-rhamnoside	C_22_H_24_O_10_	19.67	[M-H]^−^	448.4	447.6882	+	-	Ahuja, et al. (2009)
55	6-Prenylnaringenin	C_20_H_20_O_5_	27.46	[M-H]^−^	340.375	339.3389	+	+	Hitzman et al. (2020)
56	Phlorin	C_12_H_16_O_8_	29.30	[M-H]^−^	288.25	287.4053	-	+	Jungen, et al. (2020)
57	Eriodictyol	C_15_H_12_O_6_	30.38	[M-H]^−^	288.25	287.0324	+	+	Silva et al. (2010)
Anthocyanidins									
58	Peonidin 3-*O*-sophoroside	C_28_H_33_O_16_	18.48	[M+H]^+^	625.552	626.3826	+	+	Cendrowski et al. (2017)
59	Malvidin 3-*O*-(6-*p*-coumaroyl-glucoside)	C_32_H_31_O_14_	18.76	[M+H]^+^	639.580	640.3317	+	+	Degenhardt. et al. (2000)
60	Peonidin 3-*O*-(6-p-coumaroyl-glucoside)	C_31_H_29_O_13_	22.65	[M+H]^+^	609.5542	610.3495	-	+	Fontana and Schieber (2023)
61	Isopeonidin 3-O-rutinoside (tulipanin)	C_28_H_33_O_15_	22.65	[M+H]^+^	609.5526	610.3495	-	+	El Deeb, et al. (2021)
62	Pelargonidin 3-rhamnoside 5-glucoside	C_27_H_31_O_14_	26.71	[M+H]^+^	579.526	580.3821	+	-	Al-Harbi, et al. (2017)
Coumarins									
63	Scopoletin	C_10_H_8_O_4_	0.94	[M-H]^−^	192.042	191.0096	+	-	Crispim, et al. (2025)
64	4-Hydroxycoumarin	C_9_H_6_O_3_	7.14	[M+H]^+^	162.0316	162.9771	-	+	Hussein et al. (2024b)
65	Esculetin (6,7-dihydroxycoumarin)	C_9_H_6_O_4_	8.3	[M-H]^−^	178.14	176.993	+	-	Yaermaimaiti et al. (2021)
66	Daphnoretin	C_19_H_12_O_7_	13.69	[M+H]^+^	352.06	353.2978	+	-	Liliana Porras‐Dávila et al. (2023)
67	Skimmin (7-hydroxycoumarin glucoside	C_15_H_16_O_8_	18.48 18.56	[M+H]^+^ [M-H]^−^	624.282	625.4067 623.5204	+	-	Baran et al. (2024)
68	Rutaretin 9-rutinoside	C_26_H_34_O_14_	18.56	[M-H]^−^	570.539	569.7567	+	-	Villa-Jaimes et al. (2023)
69	Scopolin	C_16_H_18_O_9_	18.56	[M-H]^−^	354.308	353.1674	+	-	Cao et al. (2022)
Catechin derivatives									
70	(+)-Catechin/Epicatechin	C_15_H_14_O_6_	17.14	[M+H]^+^	290.270	291.2177	_+_	+	Ibrahim et al. (2024)
71	Gallocatechin	C_15_H_14_O_7_	18.1	[M+H]^+^	306.07	307.2134	+	-	Plumb et al. (2002)
72	Catechin 3′,4′-diglucoside	C_27_H_34_O_16_	18.56	[M-H]^−^	614.549	613.4365	+	-	Mane et al. (2022)
73	4′-methyl-(−)-epigallocatechin 3-(4-methyl-gallate)	C_24_H_22_O_11_	22.36	[M-H]^−^	486.424	485.5871	+	-	Amarowicz, et al. (2005)

#### 3.3.1 Phenolic acids

Hydroxybenzoic and hydroxycinnamic acids are common in medicinal plants and are classified by structure (Ali et al., 2020). We identified four hydroxybenzoic acid derivatives in *C. dichotoma* leaves (**1-4**): three in negative mode (**1-3**) and one in positive mode (**4**), plus one in *C. myxa* leaves (**3**). Ten hydroxycinnamic derivatives (**5**-**13**) were found in negative mode, with one (**14**) detected in both modes. Metabolites **5**, **8**, and **9** were also found in *C. myxa.* Their molecular weights and *m/z* values are presented in Table 2. In *C. dichotoma*, metabolites **1**, **2**, and **3** are syringic, protocatechuic, and vanillic acids; compound **4** is 2-hydroxybenzoic acid. Metabolite **3** was identified in *C. myxa*. Hydroxycinnamic acids from *C. dichotoma* include: **5**, latifolicinin; **6**, *p*-coumaric acid; **7**, ferulic acid; **8**, rosmarinic acid; **9**, 3-p-coumaroylquinic acid; **10**, chlorogenic acid; **11**, caffeoylquinic acid; **12**, caffeyltartaric acid; **13**, 6-*O*-*p*-coumaroyl-**D**-glucose; and **14,** *trans*-cinnamic acid. Metabolites **1**, **3**-**10**, and **14** were previously found in *C. dichotoma* leaves (Hussein H. et al., 2024; Wang et al., 1996), fruits (Hussein H. M. et al., 2024; Yaermaimaiti et al., 2021), and seeds (Yaermaimaiti et al., 2019). Compounds **3** and **8** were also identified in *C. myxa* fruits (El-Massry et al., 2021). The rest were newly identified for these species.

#### 3.3.2 Phenolic acid derivatives

Twelve phenolic acid derivatives (**15**–**26**) were found in *C. dichotoma* leaves. Compounds **15**, **16**, **19, 20**, **22**, **25**, and **26** were identified in negative mode; **17, 18, 21, 23,** and **24** in positive mode. Metabolite **23** was also observed in *C. myxa* leaves. Their molecular weights and *m/z* values are presented in Table 2. Metabolites **15** and **16** were identified as syringaldehyde and a 8*S*-(2)-8-(4-hydroxy-3-methoxy benzoyl) - dihydrofuran-8(8′H)-one. Metabolite **17** was methyl *p*-hydroxyphenyl lactate; **18** was 1-(4-hydroxyphenyl) propan-1-one. Others included compounds like 4-(3-(4-hydroxy-3-methoxy phenyl)-3-oxopropoxy)-4-oxobutanoic acid **19** and 3-(4-hydroxy-3-methoxyphenyl)-3-oxopropyl methyl succinate **20**, along with evofolin B **22**, coniferyl aldehyde **22**, hydroxybenzaldehyde **23**, 4-vinylphenol **24**, protocatechualdehyde **25**, and catechol **26**. Some metabolites (**15-22**, **25, 26**) were previously identified in *C. dichotoma* fruits (Yaermaimaiti et al., 2021), while 23 and 24 were from its seeds (Yaermaimaiti et al., 2019) and aerial parts (Bughio et al., 2024). All metabolites were first reported in *leaves of C. dichotoma and C. myxa*.

#### 3.3.3 Flavonoids

Flavonoids are widespread bioactive compounds in medicinal plants, known for their positive roles in disease treatment (Cao et al., 2022). Chemically, they feature a core structure of fifteen carbon atoms arranged in two six-membered rings linked by a three-carbon chain, forming the C6-C3-C6 configuration, and they are divided into seven subclasses (Corradini et al., 2011). In this study, 36 flavonoids were identified across both species, including 13 flavonols (9 glycosides, 4 aglycones), 11 flavones (7 glycosides, 4 aglycones), 7 flavanones (4 glycosides, 3 aglycones), and 5 anthocyanidin glycosides.

##### 3.3.3.1 Flavonols

Three flavonols (**30, 34**, **35**) were identified from *C. dichotoma* and *C. myxa*, while nine (**27-29, 31-33, and 36-38)** were only from *C. dichotoma*, and one (**39**) from *C. myxa*. Their molecular weights and *m/z* values are presented in Table 2. Metabolites **30, 34, 35**: isorhamnetin-3-*O*-rutinoside, quercetin 3-(6″-acetyl-galactoside) 7-rhamnoside, quercetin 3-(4″-acetylrhamnoside) 7-rhamnoside. *Cordia dichotoma* metabolites (**27-29, 31-33**, and 36-38): isorhamnetin, quercetin-pentoside, rutin, quercetin 4′,7-diglucoside, quercetin 7-glucuronide 3-rhamnoside, myricetin rhamnosyl-glucoside, quercetin 3-(6″-malonyl-glucoside), kaempferol-3,7-di-*O*-α-L-rhamnopyranoside, and aromadendrin. *C. myxa* metabolite **39**: taxifolin. Among these, **28** and **31**-**36** are first identified from Cordia, while **27** and **30** were previously identified in *C. dichotoma* fruits (Hussein H. M. et al., 2024; Kuppast et al., 2006). Additionally, **29**, **30**, and **38** were reported previously in *C. dichotoma* leaves (Hussein H. et al., 2024; Oza and Kulkarni, 2017; Wang et al., 1996), and **39** was identified for the first time from *C. myxa* leaves but was previously reported in the plant’s seed (Roy et al., 2014).

##### 3.3.3.2 Flavones

Nine flavones were identified from *C. dichotoma*, including apigenin 7,4′-dimethyl ether (**40**), luteolin 7-*O*-diglucuronide (**41**), 3′,5,6-trihydroxy-3,4′,7,8-tetramethoxyflavone 3-glucoside (**42**), 3′, 7,4′-trimethoxyflavone (**43**), luteolin 7-*O*-(2-apiosyl-6-malonyl)-glucoside (**44**), tricin 7-neohesperidoside (**45**), diosmin (**46**), apigenin-7-O-diglucuronide (**48**), and apigenin 7-*O*-(6″-malonyl-apiosyl-glucoside) (**49**). Moreover, as in the case of *C. dichotoma,* metabolites **41**, **45**, **46**, and **48** were identified in *C. myxa*, in addition to 5-hydroxy-3,7,4′-trimethoxyflavone 47 and luteolin 50. This is the first report confirming the presence of these flavone glycosides in both species. Notably, metabolite **43** was identified in *C. dichotoma* for the first time, and metabolites **47** and **50** in *C. myxa*.

##### 3.3.3.3 Flavanones

Five (**51-52, 54-55**, and **57)** and four **(53, 55-57)** flavanones were identified.in *C. dichotoma* and *C. myxa,* respectively. Metabolites 6-prenylnaringenin **55** and eriodictyol **57** were identified in both species. In *C. dichotoma*, metabolites **51**, **52**, and **54** were identified as naringenin-7-*O*-*β*-**D**-glucoside, hesperidin, and hesperetin-7-rhamnoside, respectively. In *C. myxa*, rhusflavanone **53** and phlorin **56** were identified. Among the identified flavanones, **51, 53, 55**, and **56** were reported for the first time from Cordia species, while **52, 54, and 57** were reported for the first time in both studied species but had been reported previously in other Cordia species (Ficarra et al., 1995; Ahuja et al., 2009; Silva et al., 2010).

##### 3.3.3.4 Anthocyanidins

Three metabolites (**58-59, 62**) and four metabolites (**58-61**) were tentatively identified in positive ion mode for *C. dichotoma* and *C. myxa*, respectively. Peonidin 3-*O*-sophoroside **58** and malvidin 3-*O*-(6-p-coumaroyl-glucoside) **59** were identified in both species. Pelargonidin 3-rhamnoside 5-glucoside **62** was only in *C. dichotoma*, wilepeonidin 3-*O*-(6-p-coumaroyl-glucoside) **60** and isopeonidin 3-*O*-rutinoside **61** were identified only in *C. myxa* leaves. All metabolites were newly identified in Cordia species.

##### 3.3.4 Coumarins

Six metabolites were identified in *C. dichotoma* (**63, 65-69**) in both negative and positive modes. They are identified as scopoletin **63**, esculetin **65**, daphnoretin **66**, skimmin **67**, rutaretin 9-rutinoside **68**, and scopolin **69**, while 4-hydroxycoumarin 64 was identified only in *C. myxa*. All tentatively identified coumarins in *C. dichotoma* are reported here for the first time in Cordia species, except **65**, previously found in the plant’s fruit (Yaermaimaiti et al., 2021). The only coumarin in *C. myxa* **64** was also first reported in this species, although it had previously been observed in *C. dichotoma* fruits (Hussein H. M. et al., 2024).

##### 3.3.5 Catechin derivatives

Four metabolites (**70** - **73**) from *C. dichotoma*, namely, (+)-catechin/(−)-epicatechin **70**, gallocatechin **71**, catechin 3′,4′-diglucoside **72**, and 4′-Methyl-(−)-epigallocatechin 3- (4-methyl-gallate) **73**. Metabolite **70** was also identified in *C. myxa.* Three of the identified catechin derivatives (**71-73**) were identified in Cordia for the first time, while 70 was previously noted in the fruits of both species (El-Massry et al., 2021; Ibrahim et al., 2024).

### 3.4 *In vivo protective effect against lung toxicity* induced *by MTX*

All biological experiments utilized a single, well-characterized batch of DAME for each species. For these batches, we documented the total phenolic content (TPC), total flavonoid content (TFC), and HPLC/MS profiles (Tables 1, 2), which serve as reference fingerprints for future standardization.

#### 3.4.1 Acute toxicity study

After administering the DAMEs from *C*. *dichotoma a*nd *C*. *myxa* and observing the mice’s behavior during the experiment, no behavioral changes were observed, and the mice survived. The DAMEs from both species are deemed safe, and the doses used in the *in vivo* study were 250 and 500 mg/kg.

#### 3.4.2 Effect of the DAME of *C. dichotoma* and *C. myxa* on the body weight

At the beginning of the experiment, body weight was similar across all groups. Conversely, on day 14, rats treated with MTX experienced a significant 21.2% reduction in body (*p <* 0.0001) compared to the control. Treatment with DEXA standard, *C. dichotoma,* and *C. myxa* DAME 250 and 500 mg/kg significantly (*p <* 0.0001) reversed the MTX-induced weight loss by 1.3-folds, 1.1-folds, 1.2-folds, 1.17-folds, and 1.2-folds, respectively. These effects did not differ significantly between the *C. dichotoma* and *C. myxa* DAME 500 mg/kg groups compared with the DEXA standard and control groups (Table 3).

**TABLE 3:** Effect of the DAME of C. *dichotoma* and C. *myxa* on the body weight.

Group	Body weight on day 0	Body weight on day 14
Control	175.6 ± 1.8	197.1 ± 1.1
MTX	175.4 ± 1.2	155.4 ± 2.3^a^
*C. Dichotoma 250*	176.3 ± 1.4	172.5 ± 0.9^a,b,c^
*C. Dichotoma 500*	175.1 ± 1.5	189.8 ± 2.4^b^
*C. Myxa 250*	176.7 ± 1.3	181.5 ± 1.7^a,b,c^
*C. Myxa 500*	176.6 ± 1.6	192.4 ± 3.4^b^
DEXA	176.4 ± 1.5	195.1 ± 2.9^b^

Data expressed as mean ± SE (n = 7). a: significantly different from the control group, b: significantly different from the MTX, group, c: significantly different from the DEXA, group. DEXA: dexamethasone, MTX: methotrexate.

#### 3.4.3 Effect of the DAME of *C. dichotoma* and *C. myxa* on oxidative stress parameters

**FIGURE 1:**
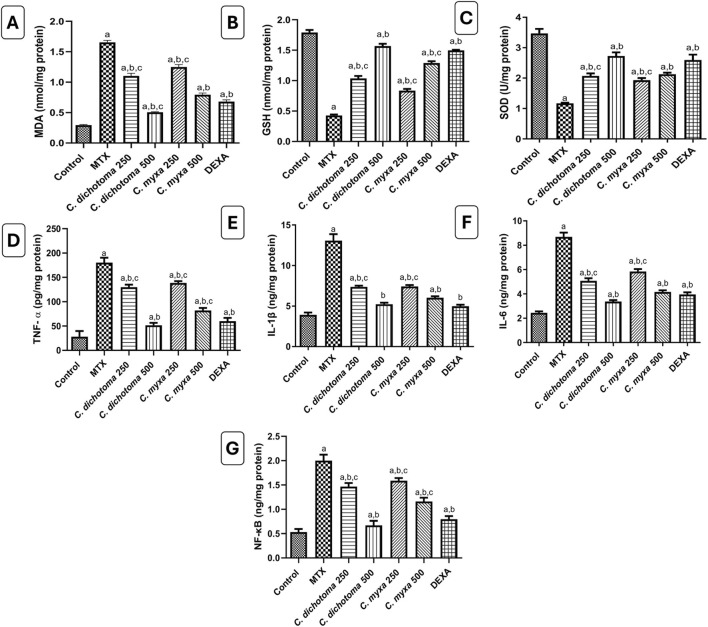
Effect of the DAME of *C. dichotoma and C. myxa* on oxidative stress and inflammatory parameters. **(A)** Malondialdehyde (MDA), **(B)** Reduced glutathione (GSH), **(C)** Superoxide dismutase (SOD), **(D**) Tumor necrosis factor–alpha (TNF-α), **(E)** Interleukin-1β (IL-1β), **(F)** IL-6, and **(G)** Nuclear factor kappa-B (NF-ĸB). Data are shown as mean ± SE (n = 7). Statistical analysis was performed using one-way ANOVA followed by Tukey’s *post hoc* test and indicated by; a: significantly different from the control group, b: significantly different from the MTX, group, c: significantly different from the DEXA, group (*p* < 0.05). DEXA: dexamethasone, MTX: methotrexate.

MTX treatment induced oxidative stress in the lung tissues, evidenced by a significant (p < 0.0001) increase in MDA levels and marked declines in GSH by 76% and in SOD by 66.3%, compared with normal rats (Figures 1A–C). Treatment with the standard, the DAME of *C. dichotoma* and *C. myxa* at 250 and 500 mg/kg significantly attenuated these alterations in a dose-dependent manner, with the highest activity recorded for *C. dichotoma* 500 mg/kg, as it significantly (p < 0.0001) decreased MDA by 69.4% and increased GSH and SOD by 3.7-fold and 1.8-fold, respectively, compared with the MTX group.

#### 3.4.4 Effect of the DAME of *C. dichotoma* and *C. myxa* on inflammatory parameters

Lung tissues from rats treated with MTX showed a significant (*p <* 0.0001) increase in TNF-α, IL-1β, IL-6, and NF-κB levels, approximately 6.4-fold, 3.3-fold, 3.6-fold, and 3.8-fold, respectively, compared with those from control rats. Treatment with standard and DAME from *C. dichotoma* and *C. myxa* at 250 and 500 mg/kg significantly (*p <* 0.0001) decreased TNF-α by 66.6%, 28%, 71.4%, 23.1%, and 54.5%, respectively; IL-1β by 61.9%, 43.6%, 60%, 43.2%, and 53.9%; IL-6 by 54.5%, 41.5%, 61.2%, 32.7%, and 52%; and NF-κB by 60.1%, 26.6%, 66.5%, 20.6%, and 42%, compared with MTX group (Figures 1D–G).

#### 3.4.5 Effect of the AME of *C. dichotoma* and *C. myxa* on fibrotic biomarkers

**FIGURE 2:**
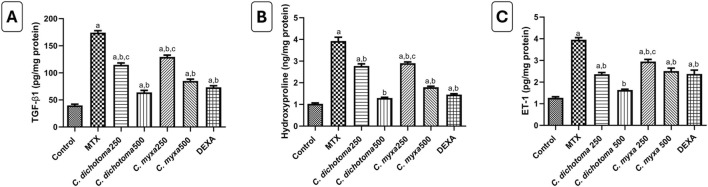
Effect of the DAME of *C. dichotoma* and C. *myxa* on fibrotic markers: **(A)** Transforming growth factor-beta (TGF-β1) and **(B)** Hydroxyproline; and **(C)** ET–1. Data expressed as mean ± SE (n = 7). Statistical analysis was performed using one-way ANOVA followed by Tukey’s *post hoc* test and indicated by. a: significantly different from the control group, b: significantly different from the MTX, group, c: significantly different from the DEXA, group (*p* < 0.05). DEXA: dexamethasone, MTX: methotrexate.

As shown in Figures 2A,B, the levels of TGF-β1 and hydroxyproline in lung tissues were significantly increased (*p <* 0.0001) in the MTX-treated group by approximately 4.4-fold and 3.8-fold, respectively, compared to the control group. Treatment with DEXA, the DAME of *C. dichotoma* and *C. myxa* at 250 and 500 mg/kg significantly (*p <* 0.0001) reduced TGF-β1 by 58%, 34.1%, 63.4%, 25.7%, and 51.2%, respectively, and hydroxyproline by 63%, 29.4%, 67.0%, 26.1%, and 54.3%, respectively, compared to the MTX-treated group. *C. dichotoma* at 500 mg/kg showed the highest activity, as it was not significantly different from the control group.

#### 3.4.6 Effect of the DAME of *C. dichotoma* and *C. myxa* on ET-1

The level of ET-1 in the MTX-treated group was significantly higher than in the control group by approximately 3.1 folds (*p <* 0.05). Treatment with DEXA and the DAME of *C. dichotoma and C. myxa* at 250 and 500 mg/kg significantly reduced ET-1 levels by about 39.9%, 40.3%, 58.6%, 25.5%, and 36.7%, respectively, compared with MTX-treated group (*p* < 0.05), as shown in Figure 2C. The most significant inhibitory effect was observed with *C. dichotoma* at 500 mg/kg, which did not differ significantly from the control.

#### 3.4.7 Effect of the DAME of *C. dichotoma* and *C. myxa* on the histopathological examination of the lung tissues

**FIGURE 3:**
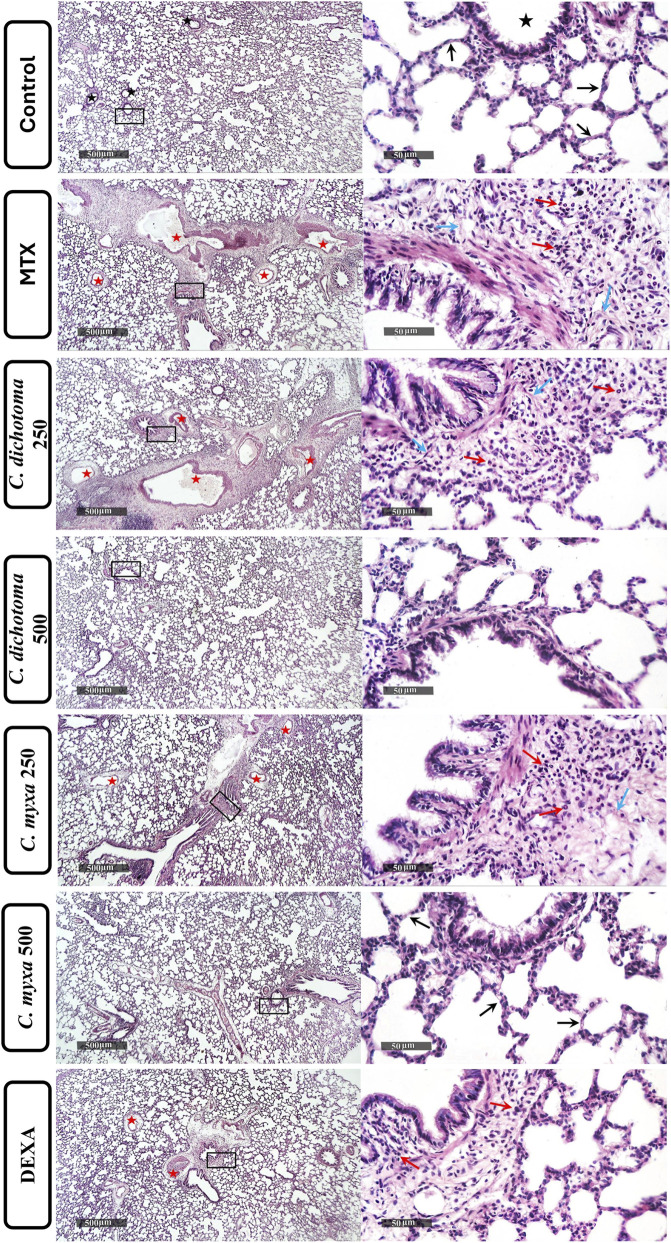
Effect of the DAME of *C. dichotoma* and *C. myxa* on the histopathological examination of the lung tissues. Microscopic examination of the control group showed normal pulmonary parenchyma, intact respiratory airways (black star), alveolar spaces, and interalveolar septa (arrow). The samples from the MTX-treated group revealed a significant increase in fibroblastic activity and collagen fibers (blue arrow), infiltration of inflammatory cells (red arrow), and multiple collapsed alveolar spaces with dilation and hyperemia of the pulmonary vasculature (red star). Samples from the *C. dichotoma* 250 mg/kg group showed minimal improvement in histological alterations of lung tissue, while the *C. dichotoma* 500 mg/kg group displayed nearly normal, organized histological features of pulmonary parenchyma, resembling those of the control group. Samples from *C. myxa* 250 mg/kg exhibited moderate fibroblastic activity, collagen fiber deposition (blue arrow), inflammatory cell infiltration (red arrow), and hyperemic vasculature (red star). The *C. myxa* 500 mg/kg group demonstrated higher dose protective effects, with almost normal organized histological features of pulmonary parenchyma and sporadic peribronchiolar inflammatory infiltrates. The standard DEXA-treated group showed decreased fibroblastic activity, mild collagen fibers around the main respiratory airways, mild peribronchiolar inflammatory cell aggregates (red arrow), and mild hyperemia of the vasculature (red star).

As shown in Figure 3, microscopic examination of the normal control group revealed a normal histological architecture of the lung parenchyma, with intact respiratory airways (black star), alveolar spaces, and interalveolar septa (arrow), free of abnormal cellular infiltration and with normal vasculature. In contrast, tissue samples from the MTX-treated group showed a significant increase in peribronchiolar and perivascular fibroblastic activity and collagen fibers (blue arrow), widespread areas of mixed inflammatory cell infiltration (red arrow), and multiple collapsed alveolar spaces throughout the lung lobules. Additionally, notable dilation and hyperemia of the pulmonary vasculature (red star) were observed. Samples from the *C. dichotoma* 250 mg/kg group showed minimal improvement in the histological alterations of pulmonary tissues. In comparison, the *C. dichotoma* 500 mg/kg group exhibited the best protective efficacy among the treated groups, with the pulmonary parenchyma showing nearly normal, well-organized histological features resembling those of the normal control group. Samples from the *C. myxa* 250 mg/kg group demonstrated moderate persistence of fibroblastic activity and collagen fiber deposition throughout the lung lobules (blue arrow). However, signs of ongoing peribronchiolar and perivascular inflammatory cell infiltration (red arrow), along with hyperemic vasculature (red star), were observed. Meanwhile, the *C. myxa* 500 mg/kg group showed relatively greater protective effects, with nearly normal, organized histological features of the pulmonary parenchyma and occasional peribronchiolar inflammatory infiltrates. The standard DEXA-treated group displayed significantly reduced fibroblastic activity, mild focal collagen fiber formation around the central respiratory airways, mild peribronchiolar inflammatory cell aggregates (red arrow), and mild vascular hyperemia (red star). There was also a higher prevalence of intact respiratory alveoli, with minimal collapsed air spaces. Semi-quantitative histopathological scoring of lung tissue alterations is demonstrated in Table 4.

**TABLE 4:** Semi-quantitative histopathological scoring of lung tissue alterations.

Groups	Inflammatory cells infiltration	Fibroblastic activity	Blood vessels dilation and hyperemia
Control	-	-	-
MTX	++++	++++	++++
*C. dichotoma* 250	++	++	++
*C. dichotoma* 500	-	+	-
*C. myxa* 250	+++	++	++
*C. myxa* 500	+	+	+
DEXA	+	+	+

Scoring system: (−) normal; (+) mild; (++) moderate; (+++) severe; (++++) very severe histological alteration.

#### 3.4.8 Effect of the DAME of *C. dichotoma* and *C. myxa* on the area percentage of collagen fibers detected by Masson’s trichrome staining

**FIGURE 4:**
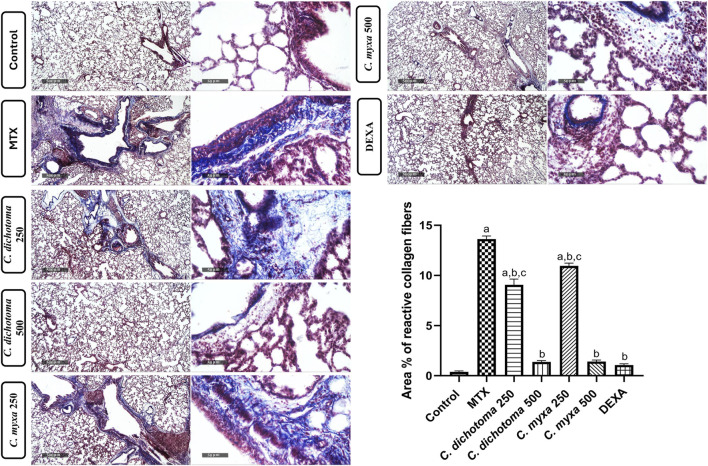
Effect of the DAME of *C. dichotoma* and *C. myxa* on the percentage area of collagen fibers detected by Masson’s trichrome staining. Data are expressed as mean ± SE (n = 7). Statistical analysis was performed using one-way ANOVA followed by Tukey’s *post hoc* test and indicated by: a: significantly different from the control group; b: significantly different from the MTX group; c: significantly different from the DEXA group (*p* < 0.05). DEXA: dexamethasone, MTX: methotrexate.

Masson’s trichrome staining of lung tissue samples of the MTX-treated group showed marked staining of collagen fibers with a significant (*P <* 0.05) increase in the area percentage of collagen fibers by 32-fold. Meanwhile, DEXA, *C. dichotoma*, and *C. myxa* at 250 and 500 mg/kg significantly decreased the percentage area of collagen fibers by 91.3%, 25.9%, 88.7%, 19.7%, and 88.4%, respectively, compared with the MTX-treated group (Figure 4).

#### 3.4.9 Effect of the DAME of *C. dichotoma* and *C. myxa* on the percentage of α-SMA positive cells measured immunohistochemically

**FIGURE 5:**
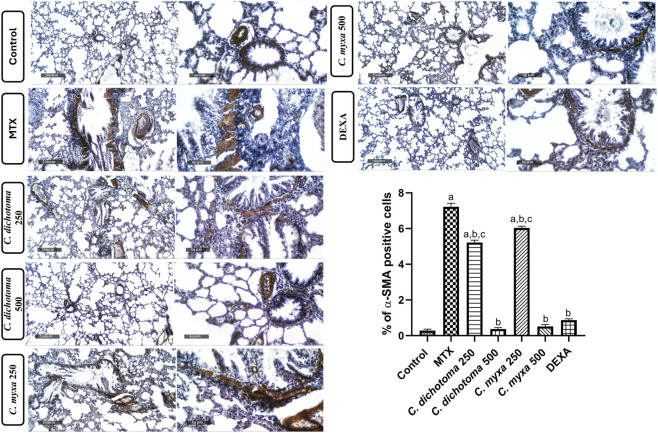
Effect of the DAME of *C. dichotoma* and *C. myxa* on the percentage of α-smooth muscle actin (α-SMA) - positive cells measured immunohistochemically. Data are shown as mean ± SE (n = 7). Statistical analysis was performed using one-way ANOVA followed by Tukey’s *post hoc* test and indicated by: a: significantly different from the control group, b: significantly different from the MTX group, c: significantly different from the DEXA group (*p* < 0.05). DEXA: dexamethasone, MTX: methotrexate.

As shown in Figure 5, lung tissues from the MTX-treated group showed a significant (*p* < 0.05) 25.5-fold increase in the percentage of α-SMA-positive cells, compared to the control group. However, lung tissues from groups treated with the AME of *C. dichotoma* and *C. myxa* at 250 and 500 mg/kg and DEXA showed a significant (*p* < 0.05) decrease in the percentage of α-SMA positive cells by 27.7%, 94.9%, 16.4%, 92.8%, and 88.0%, respectively, compared with MTX-treated group.

### 3.5 *In vitro* assay

**FIGURE 6:**
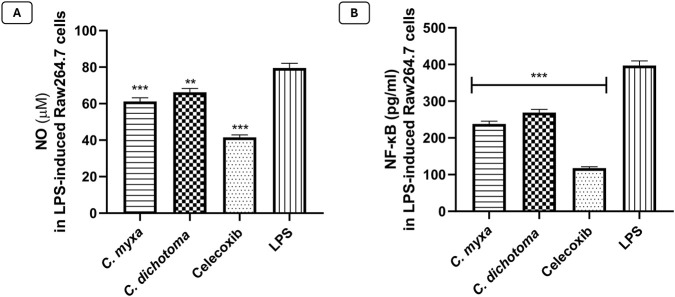
Inhibition of **(A)** Nitric oxide (NO) production and **(B)** NF-kB by the DAME of *C. dichotoma* and *C. myxa* in LPS-induced RAW cells. Data are expressed as mean ± SEM (n = 3). Statistical analysis was performed using one-way ANOVA followed by Tukey–Kramer multiple comparison test. **p < 0.01 and ***p < 0.001 versus LPS-induced untreated cells.

An MTT-based *in vitro* toxicology assay kit was used to examine the toxic effects of CD, CM, and celecoxib on RAW 264.7 cells. The IC_50_ values of CD, CM, and celecoxib were 241.15 ± 7.52, 215.75 ± 6.72, and 189.70 ± 5.53 μg/mL, respectively. LPS-activated RAW 264.7 cells were used, and NO and NF-ĸB production were measured as nitrite concentration in the culture medium. Compared with the untreated LPS control, LPS-pretreated cells released lower levels of NO into the medium, as measured by its stable, non-volatile breakdown product, nitrite. Both *C. dichotoma* and *C. myxa*, as well as celecoxib, significantly inhibited (P < 0.05) nitrite accumulation in LPS-stimulated RAW 264.7 cells (Figure 6A). The NF-ĸB level was also determined. The results showed that NF-ĸB production was increased in the culture supernatants of LPS-induced cells. These changes were markedly attenuated when RAW cells were pre-treated with *C. dichotoma*, *C. myxa*, and celecoxib (Figure 6B).

## 4 Discussion

The present study demonstrated that the extracts of *C. dichotoma* and *C. myxa* exerted a protective effect against MTX-induced lung injury, possibly through modulating an interconnected oxidative stress/inflammation/fibrosis axis. This study primarily extends the growing body of evidence supporting plant-derived polyphenols in inflammatory injury models.

MTX-induced oxidative stress functions as an upstream pathological trigger, activating redox-sensitive inflammatory pathways, particularly NF-κB signaling. Excessive oxidative stress is known to promote NF-κB activation, which in turn enhances the transcription of pro-inflammatory cytokines, thereby amplifying pulmonary inflammatory injury. Sustained inflammatory signaling subsequently activates profibrotic mediators, including TGF-β1, thereby promoting myofibroblast differentiation, α-SMA expression, and extracellular matrix deposition. Meanwhile, the observed protective effects of *C. dichotoma* and *C. myxa* suggest that attenuation of oxidative stress may suppress NF-κB-driven inflammatory cascades, thereby limiting downstream cytokine-mediated fibrotic remodeling, indicating possible antioxidant, anti-inflammatory, and antifibrotic activities.

The DAME doses used in this research (250 and 500 mg/kg) were selected based on initial toxicity assessments and previous preclinical studies involving plant extracts. To our knowledge, there is limited quantitative data on the traditional daily intake of *C. dichotoma* and *C. myxa* leaf preparations, so these doses should be regarded as experimental pharmacological levels rather than exact reflections of traditional use. Further research, including pharmacokinetic studies and human dose calculations, will be necessary to enhance the clinical applicability of these findings.

This study demonstrates that MTX induces significant oxidative and inflammatory damage in rat lungs, as evidenced by changes in oxidative biomarkers and cytokine levels. Importantly, DAME extracts from *C. dichotoma* and *C. myxa* markedly alleviated these alterations, indicating their protective potential. The 500 mg/kg dose of DAME, particularly from *C. dichotoma*, also counteracted MTX-induced body weight loss, suggesting systemic protection. MTX is widely used in oncology for autoimmune diseases (Bedoui et al., 2019). Our findings, consistent with earlier studies, indicate that oxidative stress plays a central role in MTX-induced pulmonary injury, highlighting the need for antioxidant adjuncts to mitigate its toxicity (Kahraman et al., 2013).

The interplay between oxidative stress and inflammation underlies MTX-induced lung injury (Zaki et al., 2021; Demir et al., 2024). MTX increased lipid peroxidation (MDA) while decreasing GSH and SOD, confirming redox imbalance. DAME treatment restored these parameters, reflecting its ability to enhance endogenous antioxidant defense. GSH and SOD act as the first line of defense against ROS, while excessive ROS triggers cytokine release and tissue inflammation (Ibrahim et al., 2019; El-Agamy et al., 2020; El-Sayed et al., 2023). Therefore, DAME’s antioxidant effect likely disrupts the oxidative-inflammatory cycle, thereby accounting for the observed biochemical and histological recovery.

Elevated inflammatory markers, including TNF-α, IL-1β, IL-6, and NF-κB, clearly demonstrate MTX’s inflammatory effects on lung tissue. The increase in cytokines might result from MTX’s ability to enhance the expression and secretion of these molecules, thereby increasing ROS production and lung tissue damage (Abosrea et al., 2023). The DAME treatment in both species reduced all these heightened inflammation parameters. In summary, one possible mechanism for DAME’s anti-inflammatory effect in this rat model of lung injury is a decrease in alveolar macrophage infiltration, along with lowered expression of pro-inflammatory proteins. Therefore, we suggest that DAME could help treat MTX-induced lung injury. The anti-inflammatory potential of DAME, as observed *in vivo*, is further supported by our *in vitro* findings using LPS-stimulated cells. In this model, LPS significantly increased nitric oxide production and NF-κB activation, both key mediators of inflammatory signaling. To further clarify the mechanistic link between the *in vitro* and *in vivo* findings, our results suggest that the anti-inflammatory effects observed in the MTX-induced lung injury model are, at least in part, mediated through direct modulation of macrophage activity. In LPS-stimulated RAW 264.7 cells, the extracts significantly suppressed nitric oxide production and NF-κB activation, indicating inhibition of key upstream inflammatory signaling pathways. This cellular effect is consistent with the *in vivo* findings, where reduced NF-κB levels and downstream pro-inflammatory cytokines (TNF-α, IL-1β, and IL-6), along with improved histopathological features, were observed in lung tissues. Collectively, these data support a mechanism in which attenuation of macrophage-driven NF-κB signaling contributes to the overall reduction of pulmonary inflammation and tissue injury. These findings suggest that DAME directly inhibits the NF-κB pathway, which may represent a central mechanistic event underlying the broader anti-inflammatory effects, as NF-κB is a key transcriptional regulator of cytokine production. Accordingly, suppression of NF-κB signaling is likely associated with the reduced release of pro-inflammatory mediators, thereby interrupting the self-amplifying inflammatory cycle that exacerbates tissue injury.

In addition, chronic elevation of inflammatory cytokines contributes to fibrotic progression through stimulation of profibrotic pathways such as TGF-β1 signaling (Pulito-Cueto et al., 2023). Therefore, the observed suppression of inflammatory mediators may have contributed to the attenuation of fibrosis by reducing cytokine-driven fibroblast activation and extracellular matrix accumulation. Hydroxyproline is the primary component of collagen, and changes in its levels are a key marker of collagen metabolism. During lung fibrosis, collagen accumulation is significantly increased; therefore, hydroxyproline can serve as an indicator of lung fibrosis and help determine its severity (Desaki et al., 2002). Our results showed that hydroxyproline levels were significantly reduced after DAME administration in both species, particularly at 500 mg/kg in *C. dichotoma*. Rats given daily DAME following MTX poisoning exhibited mild fibrosis around the bronchi, interstitial areas, and collagen deposits surrounding alveoli throughout the lungs. Pulmonary fibrosis is a progressive interstitial lung disorder of unknown origin, characterized by excessive accumulation of extracellular matrix in the lung interstitium. Its main stages involve inflammation and fibrosis (Pulito-Cueto et al., 2023). Numerous studies have demonstrated that TGF-β1 is a pivotal growth factor that initiates tissue repair by stimulating fibroblast proliferation and enhancing collagen gene expression, ultimately leading to tissue fibrosis (Cui et al., 2011). Our data indicate that DAME treatment reduces pulmonary collagen buildup, as evidenced by a significant decrease in lung hydroxyproline levels and lower TGF-β1 levels compared to MTX-induced lung fibrosis. Additionally, α-SMA, a significant protein in mesenchymal cells, was detected through immunohistochemical staining. α-SMA levels significantly increased in the MTX group compared to the untreated control group. Notably, DAME from both species, especially at 500 mg/kg, markedly inhibited this process and prevented the development of lung fibrosis. The downregulation of ET-1, a known mediator of fibrosis, further emphasizes the extract’s ability to interfere with key fibrotic pathways (Pulito-Cueto et al., 2023). These findings indicate that Cordia extracts not only suppress early oxidative and inflammatory processes but also prevent progression toward fibrosis, one of the most debilitating outcomes of MTX-induced lung injury. The consistency between *in vitro* and *in vivo* observations strengthens the hypothesis that these extracts target interconnected oxidative and inflammatory pathways that ultimately influence fibrotic outcomes.

Beyond biochemical measurements, histopathological examination of lungs provides a clearer, more tangible picture of how MTX damages lung tissue and how the Cordia extracts help protect against it. As expected, lung samples from the MTX-treated group showed classic features of MTX-induced pulmonary fibrosis, including collapsed alveolar spaces, inflammatory cell infiltrates, and fibrotic tissue accumulation, echoing earlier studies that have described MTX as a potent inducer of inflammatory and fibrotic damage in the lungs (Jakubovic et al., 2013; Fragoulis et al., 2019). Low doses of DAME from *C. dichotoma* and *C*. *myxa* offered modest improvement; however, high doses (500 mg/kg), particularly *C*. *dichotoma*, preserved lung architecture that closely resembled that of healthy control animals. The alveoli appeared open and well-formed, with minimal inflammation and fibrosis, paralleling the observed reduction in oxidative stress and inflammatory markers. Masson’s trichrome staining revealed an overwhelming collagen deposition in MTX-treated lung tissues, which is markedly reduced in extract-treated animals, especially with higher doses, indicating a significant reduction in fibrosis. Similarly, α-SMA staining detected a surge in myofibroblast activation after MTX treatment, indicating active fibrotic remodeling. This increase was significantly decreased upon treatment with the extracts, particularly *C. dichotoma* at higher doses by over 90%. Altogether, these histological and immunological findings reinforce the biochemical findings, indicating that Cordia extracts not only modulate the molecular signaling but also visibly preserve lung structure and prevent fibrotic events.

This study found that MTX induced degeneration of both epithelial and mesenchymal lung cells through oxidative stress and inflammation. The results showed that MTX led to lung degeneration, hyperemia, and a rise in alveolar macrophages. A notable thickening of the alveolar septal walls was frequently observed. Furthermore, the rats’ lungs showed extensive inflammation, emphysema, and necrotic cell debris in the bronchial and bronchiolar lumens. It was reported that *C*. *dichotoma* leaves extract exhibits antioxidant (Rahman and Akhtar, 2017; Hatware et al., 2018; Joshi et al., 2024) and anti-inflammatory (Hatware et al., 2018) effects, and *C. myxa* leaves and fruits also have antioxidant and anti-inflammatory properties (Abdel-Aleem et al., 2019; Samari et al., 2019). Moreover, DAME from both Cordia species mitigated these effects. HPLC/MS analysis of the two species revealed numerous phenolic metabolites, including flavonoids, phenolic acids, and their derivatives, as well as trace amounts of alkaloids and terpenoids. Polyphenols, including multiple flavonoids, are natural metabolites known for their ability to reduce inflammation and prevent oxidative stress, thereby potentially extending Lifespan. This can be achieved by inhibiting chronic inflammation signaling, which influences gene expression and leads to epigenetic changes (Luo et al., 2021).

The antioxidant and anti-inflammatory properties are strongly linked to their structural characteristics. Grasping the structure–activity relationships of phenolic metabolites is essential for understanding their mechanisms and creating more potent derivatives. Features such as multiple hydroxyl groups, especially in *ortho* positions, conjugated double bonds, and carbonyl groups enhance radical-scavenging and enzyme-inhibitory effects. Modifications such as glycosylation or methylation can significantly influence activity by altering solubility and cell penetration (De Godoy et al., 2024). The two Cordia species contain quercetin derivatives (3-OH, 4-oxo, C2 = C3, 2 *ortho* OH-group), which have been reported to exhibit antioxidant and anti-inflammatory activity by inhibiting the synthesis of inflammatory mediators (Tauchen et al., 2020) and NF-κB (Panchal et al., 2012). It also reduces extracellular matrix deposition by blocking TGF-β1 signaling in an *in vivo* model (Li et al., 2013). Furthermore, DAME contains luteolin and luteolin glycosides (4-oxo, C2 = C3, 2 *ortho* OH- group), considered potential agents for managing lung injury (Lee et al., 2010; Rungsung et al., 2018), because of their ability to downregulate pro-inflammatory mediators, inhibit MDA and NF-κB, and enhance antioxidant parameters such as SOD and GSH (Liu and Meng, 2018; Rungsung et al., 2018). Phenolic acids, such as rosmarinic acid, also possess potent antioxidant and anti-inflammatory activities due to its dual catechol groups, conjugated aromatic system, and ester linkage (El Boukhari and Fatimi, 2024). Additionally, catchine has shown antifibrotic effects in lung fibrosis, likely due to its antioxidant and anti-inflammatory effects, as well as its capacity to inhibit the activation of AKT/mTOR and Smad2/3 signaling pathways in lung tissues (Shariati et al., 2019; Wang et al., 2022). Therefore, the protective effects of DAME from *C. dichotoma* and *C. myxa* against MTX toxicity are likely linked to their polyphenolic content, which mainly exerts its effects through antioxidant and anti-inflammatory pathways. Interestingly, this study found that both extracts offered protection against MTX toxicity, but *C*. *dichotoma* showed notably higher efficacy, especially at higher doses (500 mg/kg). This may be due to its broader spectrum of bioactive metabolites, as confirmed by HPLC/MS metabolomics profiling, which includes rutin, known for its antioxidant and anti-inflammatory properties (Jia et al., 2019). Previous research has also shown that kaempferol exerts anti-inflammatory effects by inhibiting NF-κB (Qian et al., 2019) and by reducing oxidative stress and fibrotic changes (Zhang et al., 2025). Cinnamic acid has been shown to improve oxidative, inflammatory, and fibrotic parameters (Abdalhameid et al., 2024). Chlorogenic acid possesses antioxidant activity (Carrera-Cevallos et al., 2025), and synergistic acid exhibits similar properties (Jia et al., 2019). Quercetin, hesperetin, and epigallocatechin-3-gallate have also been shown to improve fibrosis (Zhang et al., 2018). Additionally, other metabolites in DAEE might contribute to the synergistic, ameliorative, and overall lung-protective effects of the two Cordia species against MTX toxicity. Based on the HPLC/MS analysis, rutin, quercetin and luteolin glycosides, rosmarinic acid, catechin derivatives, and selected coumarins are prominent constituents of the DAME preparations. These metabolites may serve as candidate markers for future quality control and standardization of Cordia leaf extracts intended for experimental or clinical use.The study is limited by the use of an acute MTX-induced fibrosis model, which may not fully mimic chronic human pulmonary fibrosis. In addition, the crude plant extracts were tested without isolation of specific active compounds, and species-related physiological differences may limit direct clinical translation. Further investigations, including bioassay-guided fractionation of the extracts to investigate the major compounds responsible for the activity, mechanistic studies, and clinical trials, are warranted to validate their potential as adjunctive therapies in MTX-treated patients.

## 5 Conclusion

This study demonstrates, for the first time, that defatted aqueous methanol extracts of *C. dichotoma* and *C. myxa* leaves exert significant protection against MTX-induced lung toxicity in rats. The observed effects are primarily linked to the extracts’ rich phenolic composition, which contributes to their antioxidant, anti-inflammatory, and antifibrotic actions. Notably, *C. dichotoma* exhibited greater efficacy, likely due to its broader profile of bioactive metabolites. Overall, this research was conducted and analyzed in accordance with the Four Pillars of Best Practice in Ethnopharmacology, emphasizing comprehensive botanical documentation, understanding of traditional use, detailed extract analysis, and clear pharmacological and toxicological evaluations. These findings highlight the preclinical protective potential of Cordia species as natural adjuvants to mitigate chemotherapy-associated lung damage. Nonetheless, additional mechanistic and clinical studies, including investigation of their active components, application in chronic models, and human-relevant dosing, are needed to bring these findings closer to practical therapeutic applications.

### 5.1 Limitation

- The study is limited by the use of an acute MTX-induced lung toxicity model, which may not fully mimic chronic human pulmonary fibrosis.
- As the findings of the study are primarily based on a preclinical animal model, in particular, the selected doses and exposure, further studies are required to incorporate pharmacokinetic profiling and estimation of human equivalent dose, to establish clinical applicability.
- The absence of deeper molecular analysis, such as gene or protein expression, beyond the NF-ĸB, limits the strength of the obtained results, suggesting that further mechanistic studies are needed
- Isolation of the major metabolites and identification of them using full spectroscopic data (^1^H and ^13^C NMR data and Mass spectroscopy).
- Species-related physiological differences may limit direct clinical translation.
- Further studies, including metabolites characterization, molecular pathway analysis, and chronic models, are needed to confirm these findings.
- Independent biological effects of the extracts in healthy animals were not specifically assessed. Future studies incorporating extract-only control groups would be valuable to distinguish baseline pharmacological actions from injury-specific protective effects.

## Data Availability

The original contributions presented in the study are included in the article/Supplementary Material, further inquiries can be directed to the corresponding authors.
