# Supplementary material for: Modulation of NF-κB and cytokine signaling by Cordia dichotoma and Cordia myxa attenuates methotrexate-induced pulmonary inflammation and oxidative injury in rats
Source: Front Pharmacol. 2026 Jun 26;17:1836019. doi: 10.3389/fphar.2026.1836019 (PMC13353095; doi:10.3389/fphar.2026.1836019)
Supplement: Supplementary file 1 [file Supplementaryfile1.docx]

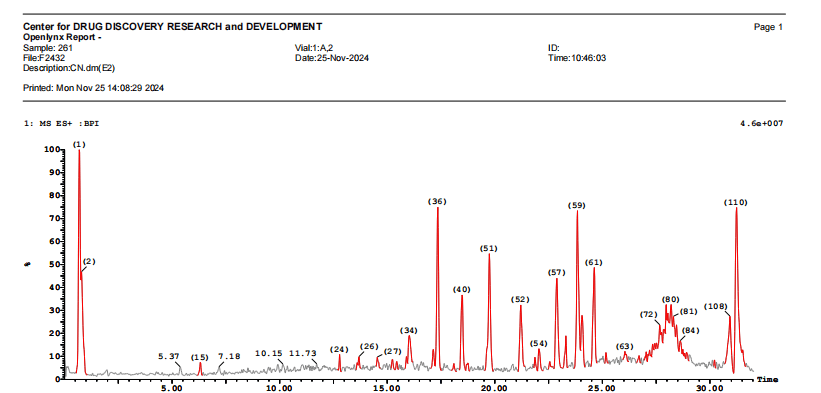


**Figure S1: Positive HPLC- ESL/MS chromatogram for *C. dichtoma* leaves**


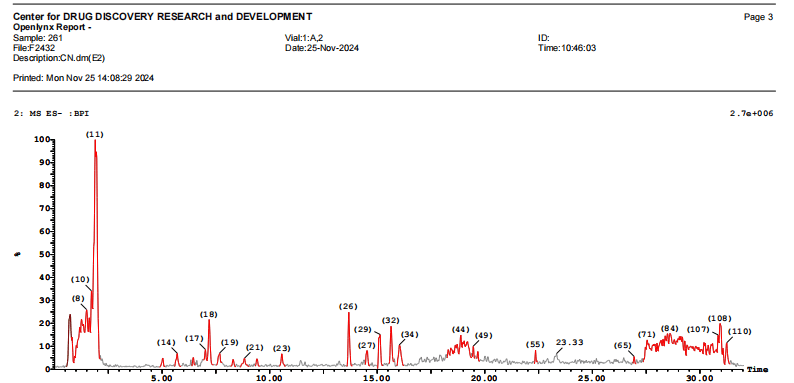


**Figure S2: Negative HPLC- ESL/MS chromatogram for *C. dichtoma* leaves**


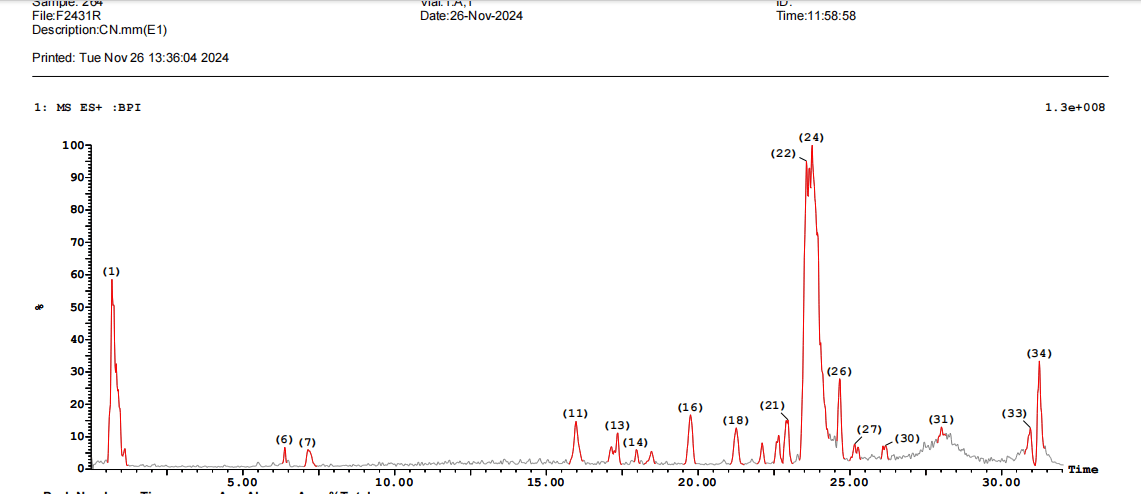


**Figure S3: Positive HPLC- ESL/MS chromatogram for *C. myxa* leaves**


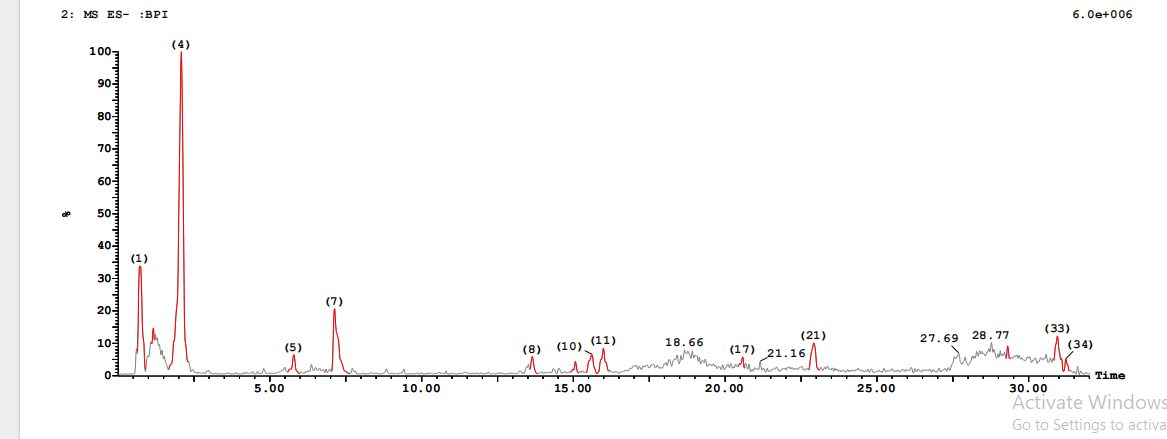


**Figure S4: Negative HPLC- ESL/MS chromatogram for *C. myxa* leaves**

| **No.** | **Compounds** | **MF** | **RT (min)** | **Mode of ionization** | **MW** | **Observed**  **(*m*/*z*)** | **CD** | **CM** | **Reference** |
| --- | --- | --- | --- | --- | --- | --- | --- | --- | --- |
| **Triterpenes** | | | | | | | | | |
|  | Cordinoic acid | C_30_H_44_O_5_ | 18.63 | [M-H] ^−^ | 484.320 | 483.4231 | + | - | Begum *et al.,* 2011 |
|  | Betulin | [C_30_H_50_O_2_](https://pubchem.ncbi.nlm.nih.gov/#query=C30H50O2) | 19.46 | [M-H] ^−^ | 442.7 | 441.3480 | + | - | Matcheme *et al*., 2025 |
|  | | | | | | | | | |
|  | Cordioic acid | C_20_H_26_O_4_ | 9.41 | [M-H] ^–^ | 330.184 | 329.3152 | + | - | Begum *et al.,* 2011 |
|  | Cordifolic acid | C_20_H_28_O_2_ | 27.46 | [M-H] ^−^ | 300.2078 | 299.0368 | + | - | Begum *et al.,* 2011 |

| **No.** | **Compounds** | **MF** | **RT (min)** | **Mode of ionization** | **MW** | **Observed**  **(*m*/*z*)** | **CD** | **CM** | **Reference** |
| --- | --- | --- | --- | --- | --- | --- | --- | --- | --- |
| **Triterpenes** | | | | | | | | | |
| **74** | Cordinoic acid | C_30_H_44_O_5_ | 18.63 | [M-H] ^−^ | 484.320 | 483.4231 | + | - | Begum *et al.,* 2011 |
| **75** | Betulin | [C_30_H_50_O_2_](https://pubchem.ncbi.nlm.nih.gov/#query=C30H50O2) | 19.46 | [M-H] ^−^ | 442.7 | 441.3480 | + | - | Matcheme *et al*., 2025 |
| **Abietane diterpenes** | | | | | | | | | |
|  | Cordioic acid | C_20_H_26_O_4_ | 9.41 | [M-H] ^–^ | 330.184 | 329.3152 | + | - | Begum *et al.,* 2011 |
|  | Cordifolic acid | C_20_H_28_O_2_ | 27.46 | [M-H] ^−^ | 300.2078 | 299.0368 | + | - | Begum *et al.,* 2011 |
| Alkaloids | | | | | | | | | |
| **774** | Isoquinolone | [C_9_H_7_N](http://www.chemspider.com/Search.aspx?q=C9H7N) | 0.79 | [M+H] + | 129.057 | 129.9696 | + | + | Chen, *et al* 2023 |
|  | Subsessiline | C_43_H_48_N_4_O_6_ | 1.34 | [M-H] ^−^ | 716.88 | 716.2165 | + | - | Goyzueta-Mamani, *et al* 2024 |
|  | 4-Hydroxyquinoline | C_9_H_7_NO | 18.63 | [M-H] ^−^ | 145.158 | 144.9228 | + | - | Zandavar and Babazad, 2023 |
| **Miscellaneous compounds** | | | | | | | | | |
| 1. **7** | Quinic acid | C_7_H_12_O_6_ | 0.69 | [M-H] ^−^ | 192.17 | 191.0671 | + | - | Hussein *et al*., 2024b |
|  | D-(+)-Malic acid | [C_4_H_6_O_5_](http://www.chemspider.com/Search.aspx?q=C4H6O5) | 0.71 | [M-H] ^−^ | 134.087 | 132.9624 | - | + | Hussein *et al*., 2024b |
|  | Ficusesquilignan A | C_31_H_36_O_11_ | 1.34 | [M-H] ^–^ | 584.225 | 583.6108 | + | - | Yaermaimaiti *et al*., 2021 |
|  | Loliolid | C_11_H_16_O_3_ | 6.31 | [M+H] + | 196.242 | 197.1066 | + | - | Yaermaimaiti *et al*., 2021 |
|  | Cordiaquinol C | C_12_H_22_O_2_ | 6.44 | [M-H] ^−^ | 246.34 | 245.0879 | + | - | Manners and Jurd,1977 |
|  | Curcasinlignan B) | C_18_H_18_O_6_ | 17.14 | [M+H] + | 330.336 | 331.2738 | + | - | Yaermaimaiti *et al*., 2021 |
|  | Cordeauxione) | C_14_H_12_O_7_ | 18.46 | [M+H] + | 292.2409 | 293.2158 | - | + | Saisin, *et al*., 2023 |
|  | Gancaonin C | C_20_H_18_O_6_ | 18.71 | [M-H] ^−^ | 354.353 | 353.3107 | + | - | Erasto *et al.,* 2004 |
|  | Piperenol A triacetate | C_27_H_26_O_10_ | 19.67 | [M-H] ^−^ | 510.489 | 509.3951 | + | - | Arena, K., *et al* 2020 |
|  | Maleic acid | C_4_H_4_O_4_ | 27.55 | [M-H] ^−^ | 116.072 | 115.7887 | + | - | Joel, *et al* 2023 |
|  | Stigmasterol | C_29_H_48_O | 27.75 | [M+H] + | 412.37 | 413.2220 | + | - | El-Di et al., *2017* |
|  | Allantoic acid | C_4_H_8_N_4_O_4_ | 31.23 | [M-H] ^−^ | 176.13 | 174.9499 | - | + | Oza and Kulkarni, 2017 |

***Triterpenes***

Cordinoic acid (74) and betulin (75) were first identified in *C. dictoma* leaves. They were detected in negative mode with *m/z* [M-H]- 483.4231 for cordinoic acid and *m/z* [M-H]- 441.3480 for betulin. Previously, these compounds were found in the stems of *C*. *latifolia* (Begum et al., 2011) and *C*. *myxa* (Matcheme et al., 2025).

***Abietane diterpenes***

Cordioic (76) and cordifolic acids (77) were first identified from *C. dictoma* leaves. They were detected in negative mode with *m/z* [M-H]- 329.3152 for cordioic acid and m/z [M-H]- 299.0368 for cordifolic acid. Both compounds had been previously found in C. *latifolia* stem bark (Begum et al., 2011).

***Alkaloids***

Three alkaloids were identified in *C. dictoma*, including isoquinolone **78 (**m/z [M+H]+ 129.9696), subsessiline **79** (m/z [M-H]- 716.2165), and 4-hydroxyquinoline (**80**, m/z [M-H]- 144.9228). Additionally, compound 80 was identified in *C. myxa*. The three compounds were newly identified from the two species, but compound **78** was also identified in *C. subcordata* (Chen et al., 2023).

***Miscellaneous compounds***

Ultimately, nine miscellaneous metabolites were detected in *C. dictoma* extract and elucidated as quinic acid **81** (*m/z* [M-H]^-^ 191.0671), ficusesquilignan A **83** (*m/z* [M-H]^-^ 583.6108), Loliolid **84 (***m/z* [M+H]^+^ 197.1066 ), cordiaquinol C **85** (*m/z* [M-H]^-^ 245.0879), curcasinlignan B **86 (***m/z* [M+H]^+^ 331.2738 ), cancaonin C **88**  **(***m/z* [M+H]^+^ 353.3107 ), piperenol A triacetate **89**

(*m/z* [M-H]- 509.3951), maleic acid **90** (*m/z* [M-H]- 115.7887), and stigmasterol **91** (*m/z* [M+H]^+^ 413.222). To our knowledge, compounds **81**, **83**, **86,** and **59** were previously identified in *C. dictoma* fruits (Yaermaimaiti et al., 2021; Hussein et al., 2024a), but were reported here for the first time from the leaves. On the other hand, malic acid **82** (*m/z* [M+H]^+^ 132.9624) and cordeauxione **87** (*m/z* [M+H]^+^ 293.2158) were identified for the first time from *C. myxa*. However, these compounds were previously identified from *C. dictoma* fruits (Hussein et al., 2024a).

Based on our previous data analysis, we found that compounds **6, 7, 8, 10, 14, 20, 29, 30, and 38** have been previously identified in *C. dictoma* leaves grown in various countries. In contrast, only one compound, **8**, was identified in *C.* *myxa.* Variability in the number of compounds identified was observed across Cordia species and other reported species, likely due to environmental factors and genetic differences. Additionally, factors such as the drying process, harvesting time, and plant age can significantly influence the chemical composition (Patel et al., 2016).

**References:**

Arena, K., Rigano, F., Mangraviti, D., Cacciola, F., Occhiuto, F., Dugo, L., et al. (2020). Exploration of rapid evaporative-ionization mass spectrometry as a shotgun approach for the comprehensive characterization of *Kigelia africana* (Lam) Benth. *Fruit. Mol. Basel Switz*. 25, 962. doi: 10.3390/molecules25040962

Begum, S., Perwaiz, S., Siddiqui, B. S., Khan, S., Fayyaz, S., and Ramzan, M. (2011). Chemical constituents of *Cordia latifolia* and their nematicidal Activity. *Chem. Biodivers*. 8, 850–861. doi: 10.1002/cbdv.201000058

Chen, Y.-L., Wang, Z.-F., Jian, S.-G., Liao, H.-M., and Liu, D.-M. (2023). Genome assembly of cordia subcordata, a coastal protection species in tropical coral islands. *Int. J. Mol. Sci.* 24, 16273. doi: 10.3390/ijms242216273

Erasto, P., Bojase-Moleta, G., and Majinda, R. R. T. (2004). Antimicrobial and antioxidant flavonoids from the root wood of *Bolusanthus speciosus*. *Phytochemistry* 65, 875–880. doi: 10.1016/j.phytochem.2004.02.011

Goyzueta-Mamani, L. D., Barazorda-Ccahuana, H. L., Candia-Puma, M. A., Galdino, A. S., Machado-de-Avila, R. A., Giunchetti, R. C., et al. (2024). Targeting Leishmania infantum mannosyl-oligosaccharide glucosidase with natural products: potential pH-dependent inhibition explored through computer-aided drug design. *Front. Pharmacol.* 15, 1403203. doi: 10.3389/fphar.2024.1403203

Joel, O., Ugwu, M., Olawuni, I., Okafo, E., and Ejike, O. (2023). Prostaglandin synthesis inhibitory activity of *Heliotropium indicum* L.(Boraginaceae) and HPLC-DAD analysis. *Tropical Journal of Natural Product Research* 7.

Manners, G. D., and Jurd, L. (1977). The hydroquinone terpenoids of *Cordia alliodora.* *J. Chem. Soc. Perkin 1*, 405. doi: 10.1039/p19770000405

Matcheme, M., Dabolé, B., Moussa, D., Nyemb, J. N., Emmanuel, T., Laurent, S., et al. (2025). Chemical constituents from *Cordia myxa* L. (Boraginaceae) and their antibacterial activity. *Nat. Prod. Res.* 39, 725–733. doi: 10.1080/14786419.2023.2288928

Patel, R. P., Singh, R., Rao, B. R. R., Singh, R. R., Srivastava, A., and Lal, R. K. (2016). Differential response of genotype × environment on phenology, essential oil yield and quality of natural aroma chemicals of five Ocimum species. *Ind. Crops Prod.* 87, 210–217. doi: 10.1016/j.indcrop.2016.04.001

Saisin, S., Panthong, K., Hongthong, S., Kuhakarn, C., Thanasansurapong, S., Chairoungdua, A., et al. (2023). Pyranonaphthoquinones and naphthoquinones from the stem bark of *Ventilago harmandiana* and their anti-HIV-1 activity. *J. Nat. Prod.* 86, 498–507. doi: 10.1021/acs.jnatprod.2c00980

Zandavar, H., and Afshari Babazad, M. (2023). “Secondary Metabolites: Alkaloids and flavonoids in medicinal plants,” in *Herbs and Spices - New Advances*, ed. E. Ivanišová (IntechOpen). doi: 10.5772/intechopen.108030
